# From fragmented neurotechnologies to closed-loop brain health systems: integrating artificial intelligence, digital health, digital twins, and advanced materials for brain disorders

**DOI:** 10.3389/fbioe.2026.1904561

**Published:** 2026-08-07

**Authors:** Ruozhu Fan, Xian Wu

**Affiliations:** 1 Graduate School, Heilongjiang University of Chinese Medicine, Harbin, Heilongjiang, China; 2 Department of Acupuncture and Moxibustion, First Affiliated Hospital of Heilongjiang University of Chinese Medicine, Harbin, Heilongjiang, China

**Keywords:** advanced biomaterials, artificial intelligence, blood–brain barrier, brain disease, digital health, digital twin, health system innovation, neurodegeneration

## Abstract

Brain disorders have traditionally been managed through fragmented clinical pathways in which diagnosis, monitoring, intervention, and long-term follow-up are treated as separate processes. This paradigm is increasingly insufficient for conditions such as Alzheimer’s disease, Parkinson’s disease, stroke, traumatic brain injury, glioma, epilepsy, and neuropsychiatric disorders, whose trajectories are dynamic, heterogeneous, and strongly shaped by biological, behavioral, environmental, and health-system factors. Recent advances in artificial intelligence, digital health, digital twin modeling, and advanced biomaterials provide an opportunity to move beyond isolated technological applications toward integrated brain health systems. In this Perspective, we argue that the major translational opportunity is not simply to apply artificial intelligence to neuroimaging, use wearable devices for neurological monitoring, or develop biomaterials for brain repair in parallel. Rather, the field should aim to build closed-loop systems in which computational models identify disease states, digital platforms continuously update patient trajectories, and advanced materials deliver adaptive therapeutic or regenerative interventions. We propose that future progress depends on three related shifts: moving from episodic diagnosis to longitudinal brain-state modeling; redefining advanced materials as programmable therapeutic interfaces rather than passive carriers; and evaluating success at the level of health-system integration rather than single-device performance. This framework may help reorient brain disease innovation from fragmented neurotechnologies toward clinically deployable, patient-specific, and dynamically adaptive brain health systems.

## 1 Introduction

Brain disorders represent one of the most complex and costly challenges facing modern medicine (Zhang and Sun, 2025). Neurodegenerative diseases, cerebrovascular disorders, traumatic brain injury, brain tumors, epilepsy, and neuropsychiatric conditions differ substantially in their pathology, but they share a common translational difficulty: they are not static diseases that can be adequately understood through isolated diagnostic events (Gao et al., 2021). They develop, fluctuate, recur, respond, and relapse across time. Yet many clinical systems continue to manage them through episodic assessments, such as a neurological examination, a single imaging scan, a short cognitive test, an electroencephalographic recording, or a limited biomarker panel (Qiu et al., 2020; Ri et al., 2022). These tools remain essential, but they provide only partial snapshots of dynamic brain states.

This snapshot-based paradigm is particularly problematic because many brain disorders evolve through long and nonlinear trajectories. Alzheimer’s disease may begin years before clinical symptoms become obvious and progresses through interactions among amyloid pathology (Therriault et al., 2023), tau accumulation, synaptic dysfunction, neuroinflammation, vascular injury, cognitive reserve, sleep, metabolic factors, and environmental exposures. Parkinson’s disease involves motor, cognitive, autonomic, psychiatric, and sleep-related manifestations that fluctuate across time and differ across individuals (Si et al., 2022; Hadjichrysanthou et al., 2020). Stroke recovery depends not only on lesion size and location, but also on neuroplasticity, rehabilitation intensity, systemic comorbidities, secondary prevention, social support, and access to follow-up care (Aswendt et al., 2021; Yan et al., 2025). Glioma management requires repeated integration of molecular diagnosis, imaging evolution, surgical feasibility, radiotherapy, chemotherapy, recurrence risk, seizure burden, neurological function, and quality of life (Li et al., 2020). In each of these examples, the central clinical challenge is not simply to assign a disease label, but to understand and influence a changing biological and functional state.

The convergence of artificial intelligence, digital health, digital twin modeling, and advanced materials offers a timely opportunity to rethink how brain disorders are diagnosed, monitored, and treated. Artificial intelligence can extract high-dimensional patterns from neuroimaging, electrophysiology, electronic health records, multi-omics data, and behavioral signals (Boyle et al., 2021; Zeng et al., 2020). Digital health technologies can extend neurological assessment beyond the hospital by capturing gait, speech, tremor, sleep, cognition, seizure events, medication adherence, rehabilitation participation, and daily functional status (Nevler et al., 2025). Digital twin models can provide patient-specific simulations of disease trajectory and therapeutic response (Aliotta et al., 2025). Advanced biomaterials can improve drug delivery across the blood–brain barrier, modulate neuroinflammation, support neural repair, or interface with neural circuits (Pardridge, 2022; Cornelison and Fadel, 2022). However, these technologies are frequently developed as parallel research tracks rather than as components of an integrated care architecture.

This separation creates a major translational gap. A highly accurate neuroimaging algorithm may not change care if it is not linked to longitudinal monitoring or treatment selection (Vinci-Booher et al., 2026). A wearable sensor may generate large quantities of data without producing actionable clinical decisions (Wang and Yang, 2025). A digital twin may simulate disease progression without being connected to real-time patient feedback (Takahashi et al., 2025). A biomaterial may deliver a therapeutic agent to the brain but remain difficult to monitor, adjust, or integrate into clinical workflows (Stein et al., 2025). As a result, the field risks producing many impressive technologies that remain fragmented, difficult to translate, and insufficiently aligned with real-world neurological care.

In this Perspective, we argue that brain disease innovation should move from fragmented neurotechnologies toward closed-loop brain health systems. The key question is no longer whether artificial intelligence can improve image interpretation, whether wearables can monitor neurological symptoms, or whether biomaterials can deliver drugs to the brain. These are important but incomplete questions. A more clinically meaningful question is whether these technologies can be connected into a system that detects brain-state changes, predicts future risk, selects or adjusts interventions, and evaluates therapeutic response over time. This shift has direct consequences for how we design studies, define endpoints, validate technologies, and judge translational readiness.

### 1.1 Positioning and novelty of this perspective

Recent reviews have summarized artificial intelligence in neuroimaging, digital biomarkers for neurological disease, wearable monitoring, brain digital twins, blood–brain barrier drug delivery, and biomaterial-based neural repair (Ghafarfaraji, 2025; Aziz et al., 2025; Maadi et al., 2021). These reviews are valuable because they map technical progress within specific domains. However, many are organized around individual technologies, disease categories, or methodological pipelines. As a result, the broader translational question remains underdeveloped: how should artificial intelligence, digital health, digital twins, and advanced materials be integrated to reshape brain disease care at the health-system level?

The present Perspective has a different purpose. Rather than offering another technique-centered review of artificial intelligence or biomaterials in neurology, we focus on a conceptual and evaluative gap that becomes increasingly important as the field moves toward clinical translation. We argue that brain disease innovation should not be judged only by the performance of isolated tools, such as the accuracy of an imaging algorithm, the sensitivity of a wearable sensor, the sophistication of a digital twin, or the loading efficiency of a nanocarrier (Sharma et al., 2021; Liu et al., 2020; Sizemore et al., 2024). Instead, success should be defined by whether these tools can contribute to a reliable, adaptive, and clinically usable system for brain-state interpretation and intervention.

The novelty of this Perspective lies in integrating three dimensions that are often discussed separately: computational brain-state modeling, digital health-enabled longitudinal monitoring, and advanced material-mediated therapeutic modulation (Xu et al., 2025; Alves et al., 2025). By linking these dimensions, we propose a closed-loop framework for brain health system innovation. This framework complements existing reviews but shifts the emphasis from technological possibility to translational architecture. In our view, the next stage of progress will depend less on optimizing artificial intelligence, digital devices, digital twins, or biomaterials in isolation, and more on determining how these technologies can be co-developed, validated, and deployed as part of patient-specific care pathways.

## 2 Brain disease innovation should target dynamic brain states, not isolated diagnostic labels

### 2.1 Diagnostic labels are necessary, but they are not sufficient for precision neurology

Neurological diagnosis has traditionally been organized around disease labels (Sar et al., 2025). This structure is clinically necessary. Patients need diagnoses, clinicians need treatment guidelines, researchers need inclusion criteria, and health systems need categories for care delivery. However, diagnostic labels alone are often insufficient for precision neurology (Bahado-Singh et al., 2023). Two patients with the same diagnosis may have very different biological mechanisms, symptom trajectories, risks, treatment responses, and rehabilitation needs. Conversely, different diagnostic entities may share overlapping pathological processes, including neuroinflammation, vascular dysfunction, mitochondrial impairment, synaptic failure, excitotoxicity, oxidative stress, and network dysconnectivity (Mata-Martínez et al., 2022).

This limitation is particularly clear in neurodegenerative disease. A patient diagnosed with Alzheimer’s disease may have mixed vascular pathology, Lewy body disease, depression, sleep disturbance, frailty, or metabolic dysfunction, all of which affect clinical progression and treatment response (Akwa, 2020; Lee et al., 2021). Similarly, Parkinson’s disease is not a single uniform disorder but a heterogeneous syndrome with variable motor phenotypes, cognitive involvement, autonomic dysfunction, psychiatric symptoms, and non-motor burden (Koros et al., 2025; Pearson et al., 2025). In stroke, the diagnostic label may identify the event but not fully describe the evolving recovery state, the risk of secondary injury, the capacity for neuroplasticity, or the patient’s rehabilitation potential (Couch et al., 2022). In glioma, molecular classification has improved precision, but management still depends on dynamic imaging changes, treatment resistance, tumor microenvironment, seizure burden, and patient functional status (Kouwenberg et al., 2022).

Artificial intelligence has begun to expose this heterogeneity by identifying patterns that are difficult to detect through conventional assessment. Multimodal models can combine neuroimaging, clinical information, neuropsychological testing, genetics, and longitudinal data to support disease subtyping or prognosis (Gao et al., 2025; Khan et al., 2025). However, if artificial intelligence is used only to assign a static label more efficiently, its transformative potential will remain limited. The more important role of artificial intelligence may be to help define dynamic brain states: measurable, updateable representations of where a patient is along a disease trajectory and how that trajectory may change under specific interventions (Rorden et al., 2025; Mathieu et al., 2026).

We therefore suggest that future brain disease innovation should move from label-centered diagnosis toward state-centered modeling. A state-centered approach does not replace clinical diagnosis; rather, it enriches it. It asks not only “What disease does the patient have?” but also “What biological and functional state is the patient currently in?”, “What trajectory is most likely?”, “Which modifiable mechanisms are active?”, and “Which intervention is most likely to change the trajectory?” These questions are better aligned with the needs of chronic, progressive, recurrent, and rehabilitation-dependent brain disorders.

### 2.2 Brain-state modeling requires multimodal and longitudinal data

A major limitation of many current artificial intelligence studies in brain disease is their reliance on cross-sectional datasets (Saxena et al., 2026; Meyer et al., 2020). Cross-sectional models can be useful for classification, but they often fail to capture how disease evolves. Brain disorders are temporal phenomena (Zhang C. et al., 2022). A single MRI scan, PET image, speech sample, gait recording, cognitive score, or seizure log may be informative, but its meaning depends heavily on prior trajectory and future change (Matejuk et al., 2024). For this reason, the next-generation of artificial intelligence in brain disease should prioritize longitudinal modeling.

Longitudinal brain-state modeling requires integration of multiple data streams. Imaging can describe structure, perfusion, metabolism, inflammation, and connectivity (Stewart et al., 2022). Electrophysiology can capture neural activity and network excitability (Peña-Ort et al., 2022). Omics data can reveal molecular vulnerability or treatment response. Digital biomarkers can reflect daily function, sleep, speech, movement, mood, cognition, and medication response (Lee et al., 2025). Clinical records can provide medication history, comorbidities, complications, hospitalizations, procedures, and care utilization (Liptzin et al., 2020). Patient-reported outcomes can capture subjective symptoms, fatigue, pain, emotional state, and quality of life (Garcia et al., 2022). Each data type is incomplete alone, but together they can form a more realistic representation of disease trajectory.

Digital health technologies are especially important because they can fill the gap between hospital visits. Many neurological symptoms fluctuate in daily life but are poorly captured during short clinical encounters. Tremor, gait freezing, sleep disruption, seizure frequency, fatigue, cognitive fluctuation, medication response, rehabilitation adherence, and social participation may all vary across hours, days, and weeks (Nham et al., 2023; Otte et al., 2020; Takeuchi et al., 2022). Wearables, smartphones, home sensors, and remote assessment platforms can convert these fluctuations into analyzable longitudinal signals. When linked with artificial intelligence models, such signals can support early warning, treatment adjustment, rehabilitation planning, relapse detection, and patient-centered follow-up (Smith et al., 2021).

However, longitudinal modeling also introduces new challenges. Data collected in real-world environments are noisy, incomplete, and influenced by device type, user behavior, social context, disease severity, caregiver support, and adherence (Ren et al., 2021). Models trained on highly curated datasets may fail in diverse populations. Clinicians may not trust predictions that are not interpretable or clinically actionable. Patients may reject monitoring systems that feel intrusive, burdensome, or disconnected from tangible care benefits. Therefore, the goal should not be data accumulation for its own sake. The goal should be clinically meaningful brain-state modeling: a system that converts multimodal longitudinal data into interpretable and actionable information.

### 2.3 Digital twins may provide a framework for individualized brain disease simulation

Digital twin technology offers a conceptual bridge between data collection and personalized intervention (Takahashi et al., 2025). A digital twin is not merely a large database or a predictive algorithm. In the context of brain disorders, it can be understood as an individualized, continuously updated computational representation of a patient’s brain structure, function, disease mechanisms, systemic physiological status, and treatment response (Zhang J. et al., 2025). Such a model could simulate future trajectories, estimate intervention effects, and support adaptive decision-making. Importantly, clinically useful brain digital twins should not be restricted to brain-intrinsic variables. Systemic comorbidities and peripheral physiological states, including cardiovascular disease, diabetes, immune dysfunction, hepatic or renal impairment, vascular risk, inflammation, drug-metabolizing capacity, and concomitant medications, may substantially influence disease progression and therapeutic behavior (Haaksma et al., 2017). These factors may shape neurological outcomes and also modify treatment exposure, drug clearance, safety monitoring, and treatment-related toxicity (Lea-Henry et al., 2018). Therefore, future brain digital twins should be developed as brain–body–therapy models that integrate neural, systemic, behavioral, and treatment-related data rather than as isolated simulations of the brain alone.

The appeal of brain digital twins lies in their ability to connect mechanistic understanding with patient-specific data. For example, a stroke recovery twin might integrate lesion location, corticospinal tract integrity, motor performance, rehabilitation intensity, wearable-derived activity, and neuroplasticity markers to predict recovery potential and guide therapy scheduling (Kim et al., 2025). A neurodegenerative disease twin might combine imaging, fluid biomarkers, cognitive tests, sleep patterns, and digital behavior to estimate progression risk and identify periods of accelerated decline (Mancini et al., 2025). A glioma twin might integrate molecular subtype, imaging growth patterns, surgical margins, radiotherapy dose, chemotherapy response, seizure burden, and patient performance status to support individualized surveillance or treatment planning (Young et al., 2020). An epilepsy twin might integrate electroencephalography, sleep, medication adherence, stress, hormonal factors, and seizure history to estimate dynamic seizure risk (Dell et al., 2021).

Nevertheless, digital twins in brain disease remain more aspirational than routine clinical reality. The brain is highly complex, and many pathological processes are incompletely understood. Patient-specific models require high-quality multimodal data, computational robustness, clinical interpretability, and prospective validation (Zeng et al., 2025). There is also a risk that the term “digital twin” becomes overused to describe any predictive model, even when the model does not truly simulate biological or clinical dynamics (Vallée, 2026). To avoid this, the field should define digital twins more strictly: they should be individualized, dynamically updated, mechanistically or physiologically informed, and connected to decision-making.

In our view, digital twin modeling should be treated as the organizing layer of closed-loop brain health systems. Artificial intelligence can generate predictions, digital health platforms can update the model over time, and advanced materials or neurotechnologies can deliver interventions. The digital twin then becomes the computational space in which diagnosis, monitoring, simulation, and therapy are integrated. This does not mean that every brain disease application needs a fully mature digital twin immediately. Rather, it suggests a direction of travel: from static algorithms toward dynamic patient-specific models that can support precision neurology.

## 3 Artificial intelligence should evolve from diagnostic automation to clinical decision architecture

### 3.1 AI in brain disease should be judged by clinical actionability, not only model performance

Artificial intelligence has achieved impressive performance in many brain disease applications, especially in neuroimaging analysis. Deep learning models can detect intracranial hemorrhage, classify brain tumors, segment stroke lesions, estimate brain age, identify structural atrophy, and support differential diagnosis in neurodegenerative disease (Otgonbaatar et al., 2025; Thamizharasi and Lakshmi, 2026). These advances are important because neurological imaging is complex, time-sensitive, and increasingly data-rich. However, high algorithmic performance does not automatically produce clinical transformation. A model may achieve strong accuracy in a retrospective dataset but fail to change diagnostic timing, treatment selection, clinical workflow, or patient outcomes (Diana-Albelda et al., 2025).

For this reason, AI tools for brain disease should be evaluated according to clinical actionability. An actionable AI system should answer a clinically relevant question, provide interpretable information, fit into the decision-making pathway, and support a specific intervention. In acute stroke, a useful model should not only identify a lesion but also support time-critical decisions about reperfusion therapy, transfer, hemorrhagic risk, or prognosis (Hope et al., 2021; Banerjee et al., 2021). In Alzheimer’s disease, a useful model should not only classify patients but also identify individuals who need further biomarker testing, risk counseling, lifestyle intervention, or disease-modifying therapy consideration (Vlachakis et al., 2021). In glioma, an AI system should not merely segment tumor volume but help integrate molecular subtype, resection planning, recurrence surveillance, and treatment response (Sill et al., 2020). In epilepsy, AI should not only detect seizures but support risk prediction, medication adjustment, and safety planning (Xu et al., 2022).

The field therefore needs a shift from “AI as diagnostic automation” to “AI as decision architecture.” Diagnostic automation emphasizes speed and accuracy for a discrete task. Decision architecture emphasizes how information is organized, interpreted, communicated, and acted upon across the patient journey. This distinction matters because brain disease care involves multiple decision points: screening, diagnosis, staging, risk prediction, treatment selection, monitoring, rehabilitation, recurrence detection, and long-term support (Altuna et al., 2025; Ghezzi et al., 2026). AI systems should be designed to connect these stages rather than optimize one isolated step.

This shift also changes how AI studies should be designed. Retrospective model development should be followed by external validation, prospective evaluation, clinician-in-the-loop testing, workflow analysis, bias assessment, and outcome-based trials. The most important question is not whether an AI model performs well under ideal conditions, but whether it improves real clinical decisions in diverse care settings.

### 3.2 Explainability is essential because brain disease decisions are high-stakes and longitudinal

Explainability is particularly important in brain disease because decisions are often high-stakes, uncertain, and longitudinal. A prediction of dementia risk may affect life planning, psychological wellbeing, insurance, and family decisions (McGrath et al., 2022). A glioma recurrence prediction may influence surgery, radiotherapy, chemotherapy, or palliative care discussions (Han et al., 2022). A stroke prognosis model may affect rehabilitation intensity and expectations (Campagnini et al., 2022). A seizure risk model may affect driving, employment, medication changes, and safety restrictions (Ghosn et al., 2023). In such contexts, clinicians and patients need more than a probability score; they need to understand why the model produced its output and how that output should be used.

Explainability should not be reduced to visual heatmaps or feature importance plots alone. These tools can be useful, but clinical explainability requires a broader framework. The model output should be linked to biologically plausible features, clinical reasoning, uncertainty estimates, and recommended actions. For example, if an AI model predicts rapid cognitive decline, it should clarify whether the prediction is driven by hippocampal atrophy, vascular burden, sleep disturbance, functional decline, biomarker profile, or prior trajectory (Etholén et al., 2025). If a stroke model predicts poor recovery, it should clarify whether this reflects lesion location, corticospinal tract injury, age, comorbidity, low activity, or insufficient rehabilitation engagement (Visvanathan et al., 2021).

Explainability also supports trust, accountability, and error correction. Clinicians are more likely to adopt AI systems when they can identify when the model is likely to be wrong. Patients are more likely to accept AI-supported care when explanations are transparent and understandable. Regulators and health systems require evidence that model outputs are robust and not biased against particular populations. Therefore, explainability is not merely a technical preference; it is a translational requirement.

In closed-loop brain health systems, explainability becomes even more important because model outputs may be repeatedly updated over time. A patient’s digital twin may change risk estimates as new imaging, wearable, or biomarker data become available (Saiz-Vivó et al., 2025). If these changes cannot be explained, clinicians may struggle to determine whether they reflect true disease progression, data noise, device error, or model drift. Future AI systems should therefore provide longitudinal explainability: not only why a prediction is made now, but why it has changed compared with the patient’s prior state.

### 3.3 Foundation models and multimodal AI may reshape brain health systems, but require careful validation

The rapid development of foundation models and multimodal AI creates new opportunities for brain disease research. These models may integrate imaging, text, structured clinical data, genomics, digital biomarkers, and patient-reported information within a shared computational framework (Tavakoli et al., 2025). In principle, they could help summarize complex patient histories, identify hidden disease patterns, support clinical documentation, generate personalized risk profiles, and coordinate care recommendations. In brain disease, where information is distributed across imaging reports, neurological examinations, cognitive tests, medication records, EEG data, rehabilitation notes, and caregiver observations, multimodal AI could be especially valuable (Topol, 2020).

However, the use of foundation models in brain health should be approached cautiously. General-purpose models may produce plausible but inaccurate outputs, fail to capture domain-specific uncertainty, or reproduce biases present in training data. Brain disease care often involves vulnerable patients, ambiguous symptoms, and high-stakes decisions (Gu et al., 2022). Therefore, foundation models should not be deployed as autonomous decision-makers. Their role should be carefully defined as assistive, interpretable, auditable, and clinician-supervised.

A promising direction is the development of domain-specific multimodal brain models. Such models could be trained or adapted using neuroimaging, electrophysiology, clinical notes, disease registries, digital biomarker streams, and molecular data (Lu et al., 2025). They could serve as computational engines within closed-loop systems, helping integrate heterogeneous data into patient-specific state representations (Serag et al., 2025). However, their clinical value will depend on rigorous validation across institutions, devices, languages, populations, and disease stages.

We suggest that multimodal AI should be evaluated not simply by benchmark performance but by its contribution to system-level care. Does it reduce diagnostic delay? Does it improve patient stratification? Does it support more appropriate treatment selection? Does it reduce clinician burden? Does it detect deterioration earlier? Does it improve patient or caregiver experience? These questions will determine whether multimodal AI becomes a transformative component of brain health systems or remains a technically impressive but clinically peripheral tool.

## 4 Digital health should evolve from remote monitoring to therapeutic infrastructure

### 4.1 Wearables and mobile platforms can extend neurological assessment beyond the clinic

Digital health has already begun to change neurological assessment by enabling remote and continuous data collection (Nainwal et al., 2025). Wearable sensors can capture gait, tremor, balance, sleep, physical activity, heart rate variability, and fall risk (Miller et al., 2022). Smartphones can record speech, typing behavior, cognition, social interaction, medication adherence, and geolocation-derived mobility patterns. Home-based systems can monitor seizure events, rehabilitation exercises, daily function, environmental exposures, and caregiver-reported changes (Myers et al., 2025). These tools are particularly valuable because many neurological symptoms are intermittent, context-dependent, or underreported during clinic visits.

In Parkinson’s disease, wearable sensors may detect motor fluctuations, dyskinesia, bradykinesia, gait instability, and response to dopaminergic therapy (Moon et al., 2020). In Alzheimer’s disease and mild cognitive impairment, passive digital markers may capture changes in mobility, sleep, communication, or daily routines before overt clinical decline becomes obvious (Bachmann et al., 2023). In epilepsy, seizure detection and prediction tools may improve safety and reduce uncertainty (Narodova, , 2025). In stroke rehabilitation, sensor-based monitoring can quantify real-world limb use and activity levels, which may differ substantially from performance in supervised therapy sessions (Xie et al., 2025). In traumatic brain injury, digital tools may track sleep, headache, cognitive fatigue, mood, and gradual return to activity (Johansson, 2021).

However, remote monitoring alone does not automatically improve outcomes. A large volume of data may overwhelm clinicians if it is not filtered, interpreted, and linked to action. Patients may lose interest if monitoring does not produce visible benefits. False alarms may reduce trust. Device differences may limit comparability. Socioeconomic disparities may affect access and adherence. Therefore, digital health must be designed as therapeutic infrastructure rather than simply as data collection.

Therapeutic infrastructure means that digital tools should support decisions, interventions, and feedback loops. A wearable system should not merely report that a patient walks less; it should help determine whether reduced mobility reflects disease progression, depression, medication side effects, environmental barriers, or rehabilitation failure (Zhao et al., 2020). A cognitive app should not merely generate scores; it should help identify patterns that require clinical evaluation or intervention (Nakaoku et al., 2025). A seizure monitoring system should not merely count events; it should support risk prediction, medication adjustment, and safety planning. This is the level at which digital health becomes clinically meaningful (Wilms et al., 2026).

### 4.2 Digital biomarkers must be clinically interpretable and biologically anchored

The rapid expansion of digital biomarkers has created enthusiasm, but also conceptual risk. Not every measurable digital signal is a clinically meaningful biomarker. A digital biomarker should be reliable, interpretable, biologically or functionally relevant, and linked to decision-making. In brain disorders, this requirement is especially important because digital signals may reflect multiple interacting factors. A change in gait may indicate Parkinsonian progression, stroke recovery failure, medication effect, fatigue, pain, depression, or environmental change (Felius et al., 2025). A change in speech may reflect neurodegeneration, mood disturbance, sleep deprivation, or device-related recording variability. A change in activity may reflect neurological decline, weather, social isolation, musculoskeletal pain, or caregiver availability (Prenkaj et al., 2023).

Therefore, digital biomarkers should be biologically anchored. This does not mean that every digital signal must correspond to a single molecular mechanism. Rather, it means that the relationship between the digital measure and the clinical or biological state should be explicitly defined. For example, wearable-derived gait asymmetry after stroke should be linked to motor impairment, balance, activity participation, and rehabilitation goals. Speech changes in Parkinson’s disease should be linked to motor control, cognition, and daily communication (Gao et al., 2024). Passive behavioral markers in Alzheimer’s disease should be linked to functional decline, cognitive status, caregiver-relevant outcomes, and safety (Rabbito et al., 2020). Sleep-related signals should be interpreted in relation to neurodegeneration, mood, circadian rhythm, medication, and comorbidity (Topalidis et al., 2025).

Artificial intelligence can help identify complex patterns in digital biomarker data, but interpretability remains essential. Clinicians need to know what a model’s output means and what action should follow. Patients need to understand why they are being monitored and how the information benefits their care. Regulators need evidence that digital biomarkers are valid, robust, and clinically useful. Without such evidence, digital biomarker systems may remain research tools rather than components of routine care.

In our view, digital biomarkers should be evaluated according to three levels. The first level is technical validity: does the device measure the signal reliably? The second is clinical validity: does the signal reflect a meaningful disease state or functional outcome? The third is clinical utility: does the signal improve decision-making, treatment adjustment, patient safety, trial efficiency, or health-system performance? Many studies stop at the first or second level. Closed-loop brain health systems require all three.

### 4.3 Digital platforms should connect patients, clinicians, algorithms, and interventions

The full value of digital health emerges when platforms connect multiple stakeholders and technologies. In a closed-loop brain health system, patients generate real-world data through devices and self-reports; clinicians interpret clinically relevant summaries; artificial intelligence models update risk estimates; digital twins simulate possible trajectories; and therapeutic interventions are adjusted accordingly (Graf et al., 2025). Such platforms could support personalized medication management, rehabilitation scheduling, neuromodulation tuning, biomaterial therapy follow-up, recurrence surveillance, and long-term prevention.

This vision is especially relevant for chronic brain disorders that require continuous management. For example, a patient with Parkinson’s disease may benefit from a platform that integrates medication timing, wearable motor data, sleep quality, mood symptoms, fall risk, and clinician review (Daneault et al., 2021). A stroke survivor may benefit from a platform that combines home exercise data, gait metrics, blood pressure, depression screening, and rehabilitation goals (Germanotta et al., 2022). A glioma patient may benefit from integration of imaging follow-up, molecular risk, treatment toxicity, cognitive status, seizure burden, and quality-of-life monitoring (Yan et al., 2021). A patient with epilepsy may benefit from a platform that links seizure detection, sleep quality, medication adherence, stress, menstrual cycle, and individualized risk alerts (Busia et al., 2025).

Nevertheless, platform design must avoid excessive technological burden. Brain disease patients may have cognitive impairment, motor limitations, fatigue, visual problems, aphasia, psychiatric symptoms, or caregiver dependence (Segato et al., 2020). Digital tools must therefore be accessible, low-friction, and adaptable to patient ability. Data governance is also central. Brain health data may include sensitive behavioral, cognitive, emotional, and functional information (Paul et al., 2022). Ethical deployment requires transparency, consent, privacy protection, cybersecurity, and clear accountability for algorithm-informed decisions (Bhasha and Panda, 2024).

We suggest that future digital platforms for brain disease should be judged by integration capacity. A platform that collects data but does not inform action is incomplete. A platform that provides predictions but cannot fit into clinical workflow is unlikely to be adopted. A platform that supports clinicians but burdens patients may fail in practice. The most valuable systems will be those that connect data, interpretation, intervention, and follow-up in a way that is clinically meaningful, ethically acceptable, and sustainable.

## 5 Advanced materials should become programmable therapeutic interfaces, not passive delivery vehicles

### 5.1 The blood–brain barrier makes material innovation central to brain disease therapy

The blood–brain barrier remains one of the most important obstacles in the treatment of brain disorders. Many potentially effective therapeutic agents fail because they cannot reach the brain at sufficient concentrations without systemic toxicity. This problem affects neurodegenerative disease, brain tumors, stroke, traumatic brain injury, epilepsy, and neuroinflammatory disorders. It is therefore unsurprising that nanocarriers, hydrogels, exosomes, polymeric systems, lipid nanoparticles, cell-membrane-coated particles, and stimuli-responsive materials have become major areas of investigation (Ma et al., 2025).

However, the translational value of brain-targeted materials should not be judged only by whether they cross the blood–brain barrier. Delivery is necessary, but it is not sufficient. A clinically useful material system must deliver the right cargo to the right region at the right time, at a safe dose, while responding appropriately to disease microenvironments and avoiding unacceptable immune, vascular, or off-target effects. In brain tumors, for example, the target is not simply “the brain” but a heterogeneous tumor microenvironment with variable permeability, hypoxia, immune suppression, infiltrative margins, and therapy-resistant cell populations (Tonello et al., 2025). In stroke or traumatic brain injury, the therapeutic window changes rapidly across acute injury, inflammation, repair, and remodeling (Bosak et al., 2022). In neurodegeneration, long-term safety and repeated administration become central concerns.

This means that advanced materials for brain disease should increasingly be designed as programmable therapeutic interfaces (Zhang H. et al., 2022). A programmable interface is not a passive carrier. It can respond to pathological cues, release therapeutics in a controlled manner, modulate local microenvironments, interact with cells or neural circuits, and potentially communicate with external monitoring or stimulation systems. For instance, materials may be engineered to respond to pH, oxidative stress, enzymes, inflammatory mediators, magnetic fields, ultrasound, light, or electrical stimulation (Liu et al., 2025). In a practical closed-loop design, local biological signals could be captured by sensing elements embedded within or positioned adjacent to the material, such as electrochemical, optical, mechanical, enzyme-responsive, antibody- or aptamer-functionalized, or conductive polymer components. These signals could then be converted into readable outputs, including electrical current, optical signals, magnetic responses, impedance changes, or imaging-detectable contrast, and transmitted to an external digital platform through wireless microelectronics, near-field communication, wearable or bedside readers, or clinically available imaging and stimulation systems (Scholten and Meng, 2018; Zheng et al., 2024). In the near term, such functions are more likely to be achieved through hybrid material–bioelectronic interfaces rather than by passive biomaterials alone. Such responsiveness and signal communication may allow therapies to be spatially and temporally aligned with disease activity.

The implication for the field is clear: material design should be guided by brain-state information. If artificial intelligence and digital monitoring can identify when inflammation, seizure risk, tumor recurrence, or neurodegenerative acceleration is likely, then material-based therapies should eventually be designed to respond to these states. This shifts biomaterials from the role of “delivery platforms” to the role of active therapeutic participants within a closed-loop system.

### 5.2 Regenerative biomaterials must be evaluated by functional integration, not only histological repair

Biomaterials for neural repair have often been evaluated through structural or histological endpoints: reduced lesion cavity, increased neuronal marker expression, enhanced axonal growth, decreased glial scarring, or improved tissue organization (Raspa and Gelain, 2021). These endpoints are important, but brain repair is not simply a matter of filling a defect or increasing marker expression. The nervous system is defined by connectivity, plasticity, electrical activity, and behavioral output (Shen, 2022). Therefore, regenerative biomaterials for brain disease should ultimately be evaluated by whether they support functional integration.

This issue is especially relevant for stroke and traumatic brain injury. A scaffold or hydrogel may create a permissive microenvironment for cell survival or axonal extension, but functional recovery depends on whether new or spared neural circuits can support meaningful behavior (Zheng et al., 2026). Similarly, conductive or electroactive materials may promote neural activity, but their clinical relevance depends on whether they can integrate safely with host networks and support controlled plasticity rather than aberrant excitation. In neurodegenerative disease, regenerative strategies must also confront a hostile chronic environment characterized by inflammation, protein aggregation, vascular dysfunction, mitochondrial impairment, and progressive network failure (Hu et al., 2021).

For these reasons, biomaterial studies in brain disease should adopt more demanding and operationally defined evaluation frameworks. Histology should be combined with electrophysiology, imaging, behavioral assessment, circuit-level analysis, inflammatory profiling, and longitudinal monitoring. One practical approach is to organize functional integration into a prespecified, multi-level assessment framework. Structural and histological readouts can first determine whether the material supports tissue repair or reduces lesion-related damage. Circuit-level and physiological readouts, such as EEG changes, evoked potentials, local field potentials, or functional connectivity, can then assess whether structural repair is accompanied by restoration of neural activity (Keser et al., 2022). Task-based and clinical functional readouts, including motor performance, cognitive scores, language recovery, seizure burden, or neurobehavioral scales, can evaluate whether these changes translate into measurable functional benefit. Real-world digital readouts, such as wearable-derived gait metrics, limb use, activity levels, and daily functional patterns, can provide longitudinal evidence of whether the intervention improves function outside the laboratory or clinic (Mohan et al., 2021). These readouts should be selected according to the disease context and therapeutic goal, with prespecified primary and secondary functional endpoints rather than an unstructured collection of measurements. In preclinical studies, short-term structural improvement should not be interpreted as sufficient evidence of meaningful repair. In translational studies, outcomes should include safety, durability, functional performance, quality of life, and compatibility with rehabilitation or pharmacological treatment.

A closed-loop framework could make this evaluation more rigorous. Digital biomarkers may detect changes in gait, limb use, speech, cognition, seizure burden, sleep, or daily function after material-based intervention (Mancini et al., 2025). Imaging and electrophysiology may reveal whether structural repair corresponds to network recovery (Gugger et al., 2023). Artificial intelligence may integrate these outcomes to distinguish true functional improvement from transient compensation or measurement noise (Segato et al., 2020). In this sense, digital health and artificial intelligence are not only diagnostic tools; they may become essential for evaluating whether biomaterials actually improve brain function.

### 5.3 Material development should be co-designed with computational prediction and clinical workflow

Many biomaterial systems fail to translate because they are developed under laboratory conditions that do not reflect clinical workflows. A brain-targeted nanocarrier may show excellent uptake *in vitro* but be difficult to manufacture reproducibly (Wang et al., 2020). A hydrogel may improve repair in a small-animal model but be unsuitable for human-scale lesions (Lotz et al., 2021). A stimuli-responsive system may require activation methods that are impractical, invasive, or unsafe in clinical settings. A regenerative construct may work biologically but fail regulatory, sterility, storage, or delivery requirements.

To address this gap, material development should be co-designed with computational prediction and clinical workflow from the beginning. Artificial intelligence can support material discovery by predicting structure–property relationships, optimizing drug loading, estimating release kinetics, modeling blood–brain barrier transport, and identifying patient subgroups most likely to benefit (Suvarna et al., 2025). Digital twin models can simulate where, when, and how a material-based intervention may alter disease trajectory (Wen, 2025). Clinical workflow analysis can determine whether a material can be administered during surgery, delivered intravenously, injected locally, activated noninvasively, monitored remotely, and repeated safely.

This co-design logic is particularly important for brain diseases because the margin for error is narrow. The brain is anatomically and functionally sensitive, and adverse effects may be severe. Therefore, translational readiness should include not only efficacy but also controllability, reversibility, long-term safety, monitoring capacity, and compatibility with existing neurological care pathways. For chronic brain diseases, long-term safety should be treated as a design requirement rather than only a late-stage evaluation endpoint. Key issues include whether material degradation kinetics match the disease course and therapeutic window, whether degradation products or non-degradable components accumulate in brain tissue or peripheral organs (Perrigue et al., 2021), whether repeated administration or long-term implantation triggers chronic inflammation, glial responses, fibrotic encapsulation, or immune recognition (Anderson et al., 2008), and whether material status and local tissue responses can be monitored over years through imaging, implantable sensing, biochemical readouts, or digital follow-up. A material that cannot be monitored after administration may be less attractive than one whose therapeutic effects and safety profile can be tracked over time. Likewise, a material that cannot be adjusted in response to disease evolution may be less suitable for chronic disorders than one designed for adaptive or staged therapy.

We propose that future studies should treat computational tools and material platforms as interdependent. Instead of asking whether artificial intelligence can predict outcomes after a material is developed, investigators should ask how artificial intelligence can shape material design before experiments begin. Instead of asking whether digital monitoring can be added after therapy, they should ask how monitoring endpoints can be built into the therapeutic concept itself. This shift would bring brain biomaterials closer to the health-system innovation goals of precision medicine.

## 6 Disease-specific scenarios for closed-loop brain health systems

### 6.1 Alzheimer’s disease and related dementias: from late diagnosis to trajectory modification

Alzheimer’s disease and related dementias provide a clear example of why fragmented care is insufficient. Traditional diagnosis often occurs after meaningful cognitive and functional decline has already emerged. Even when imaging, cerebrospinal fluid biomarkers, or blood-based biomarkers improve early detection, patients still require long-term monitoring, risk stratification, treatment planning, caregiver support, and functional follow-up (Varma et al., 2023). A closed-loop brain health system could integrate cognitive testing, neuroimaging, blood biomarkers, sleep, physical activity, speech, medication history, vascular risk, and passive digital behavior to model individual trajectories (Li et al., 2025).

In this framework, artificial intelligence would not simply classify Alzheimer’s disease *versus* normal aging. It would estimate the patient’s current brain state, identify modifiable risk factors, predict decline speed, and support decisions about further testing or treatment (Song et al., 2025). Digital health tools could detect changes in daily routines, sleep, mobility, communication, and functional independence (Cowie and Lam, 2021). A digital twin could simulate how vascular control, sleep improvement, cognitive intervention, medication, or disease-modifying therapy might alter trajectory (Awasthi et al., 2025). Advanced materials may eventually contribute through targeted delivery of anti-inflammatory, neuroprotective, gene-regulatory, or protein-clearance strategies.

The key translational endpoint should not be diagnostic accuracy alone. A clinically meaningful system should improve earlier recognition, reduce uncertainty, support personalized counseling, guide intervention timing, monitor safety, and preserve function. It should also address caregiver burden, because dementia care depends heavily on family and social support. In this disease context, the closed-loop model emphasizes longitudinal care rather than one-time diagnosis.

### 6.2 Parkinson’s disease: from symptom scoring to adaptive motor and non-motor management

Parkinson’s disease is well suited to digital health integration because many of its symptoms fluctuate across time. Motor fluctuations, dyskinesia, freezing of gait, tremor, bradykinesia, sleep disturbance, autonomic symptoms, mood changes, and cognitive decline may not be fully captured during clinic visits (Antar et al., 2021). Wearables, smartphones, and home-based monitoring can provide continuous assessment of motor and non-motor states. Artificial intelligence can integrate these signals with medication timing, clinician ratings, patient-reported outcomes, and environmental factors (Lopes et al., 2025).

A closed-loop Parkinson’s system could support adaptive medication management, fall prevention, rehabilitation planning, and neuromodulation tuning. For patients receiving deep brain stimulation, digital signals and AI models may help adjust stimulation parameters based on symptom state (Fedotchev et al., 2021). For patients at earlier stages, gait, tremor, speech, and sleep markers may help detect progression or treatment complications. A digital twin could simulate symptom patterns and expected response to medication or stimulation changes (Vallée, 2026). Advanced materials may contribute through neuroprotective delivery systems or regenerative approaches targeting dopaminergic pathways, although these remain translationally challenging.

The major conceptual shift is from episodic symptom scoring to adaptive disease-state management. Parkinson’s disease is not adequately represented by a single clinic-based motor score (Takiyama et al., 2020). A closed-loop approach would treat the patient’s functional state as dynamic and context-dependent. Success should be measured by improved symptom control, reduced falls, better daily function, improved quality of life, reduced caregiver burden, and more efficient clinical decision-making (Xiang et al., 2025).

### 6.3 Stroke: from acute lesion detection to recovery-state optimization

Stroke care has already benefited from imaging, artificial intelligence, and systems-level coordination, especially in acute triage and reperfusion decision-making (Rondina and Nachev, 2025). However, stroke is not only an acute vascular event; it is also a long-term recovery process. Many patients experience persistent motor impairment, aphasia, cognitive deficits, depression, fatigue, spasticity, pain, and reduced social participation (Mourid et al., 2020). The current care pathway often separates acute hospital treatment from rehabilitation and long-term prevention, creating gaps in recovery monitoring and secondary prevention.

A closed-loop stroke system would integrate acute imaging, lesion location, vascular risk, treatment timing, rehabilitation data, wearable-derived movement, speech recovery, mood, blood pressure, medication adherence, and patient-reported outcomes (Hays et al., 2026). Artificial intelligence could estimate recovery potential and risk of complications (Bier et al., 2025). A digital twin could simulate rehabilitation trajectories and help determine therapy intensity, timing, and modality. Digital platforms could monitor real-world limb use, gait, falls, speech practice, and home exercise adherence (Coates et al., 2020). Advanced biomaterials may contribute to neuroprotection, inflammation modulation, local drug delivery, or neural repair, particularly if paired with rehabilitation and digital follow-up.

The central endpoint should shift from lesion detection to recovery-state optimization. Acute treatment remains critical, but long-term outcomes depend on how the health system supports neuroplasticity, function, and prevention after discharge. A closed-loop model could help align hospital care, rehabilitation, home monitoring, and secondary prevention into a continuous pathway (Eyal, 2025).

### 6.4 Glioma and other brain tumors: from static imaging follow-up to adaptive oncological neurocare

Glioma management is inherently dynamic. Diagnosis, surgery, radiotherapy, chemotherapy, targeted therapy, imaging surveillance, recurrence assessment, seizure control, neurological function, cognition, and quality of life must be integrated across time (Kouwenberg et al., 2022). Current follow-up often depends heavily on periodic imaging and clinical examination. However, tumor progression, treatment-related changes, pseudoprogression, radiation necrosis, seizure burden, cognitive decline, and therapy toxicity may be difficult to distinguish (Quan et al., 2021).

A closed-loop brain tumor system could integrate radiomics, molecular pathology, surgical data, treatment exposure, longitudinal imaging, seizure logs, neurocognitive testing, steroid use, digital functional status, and patient-reported outcomes (Ling et al., 2026). Artificial intelligence could support segmentation, progression assessment, recurrence prediction, and treatment response evaluation. A digital twin could simulate tumor growth and treatment effects (Digital Twin, 2025). Advanced materials may contribute through local drug delivery, implantable depots, blood–brain barrier modulation, immune microenvironment regulation, or device-assisted delivery strategies.

In this context, success should not be defined only by imaging accuracy or drug delivery efficiency. It should include survival, neurological function, cognitive preservation, seizure control, quality of life, treatment burden, and decision support. A closed-loop system could help neuro-oncology move from periodic surveillance toward adaptive oncological neurocare.

### 6.5 Epilepsy and traumatic brain injury: From event documentation to risk-state prediction

Epilepsy and traumatic brain injury illustrate the importance of risk-state prediction (Pease et al., 2022). In epilepsy, seizures may be intermittent and difficult to predict, but their consequences can be severe. Digital tools can capture seizure events, sleep, stress, medication adherence, heart rate, movement, and electrographic signals. Artificial intelligence may help estimate periods of increased seizure risk (Jeppesen et al., 2020). A digital twin could integrate individual triggers, medication response, and longitudinal event patterns. Closed-loop neuromodulation already reflects this logic at the device level, but broader digital integration could extend it to patient-centered management (Ling et al., 2026).

Traumatic brain injury also evolves across time. Acute injury may be followed by neuroinflammation, cognitive impairment, sleep disturbance, mood disorders, headache, endocrine dysfunction, and neurodegenerative risk (Almarghalani et al., 2023). A closed-loop system could integrate imaging, symptom reporting, sleep, activity, cognitive testing, mood, and rehabilitation progress (Zhang M. et al., 2022). Advanced materials may eventually contribute to neuroprotection, inflammation modulation, or repair, but their value will depend on careful monitoring and functional outcome assessment.

For both epilepsy and traumatic brain injury, the goal should shift from documenting events after they occur to identifying risk states before harm accumulates. Such a shift requires reliable sensing, interpretable prediction, actionable intervention, and ethical data governance.

## 7 Toward closed-loop brain health systems

### 7.1 The proposed framework

**FIGURE 1:**
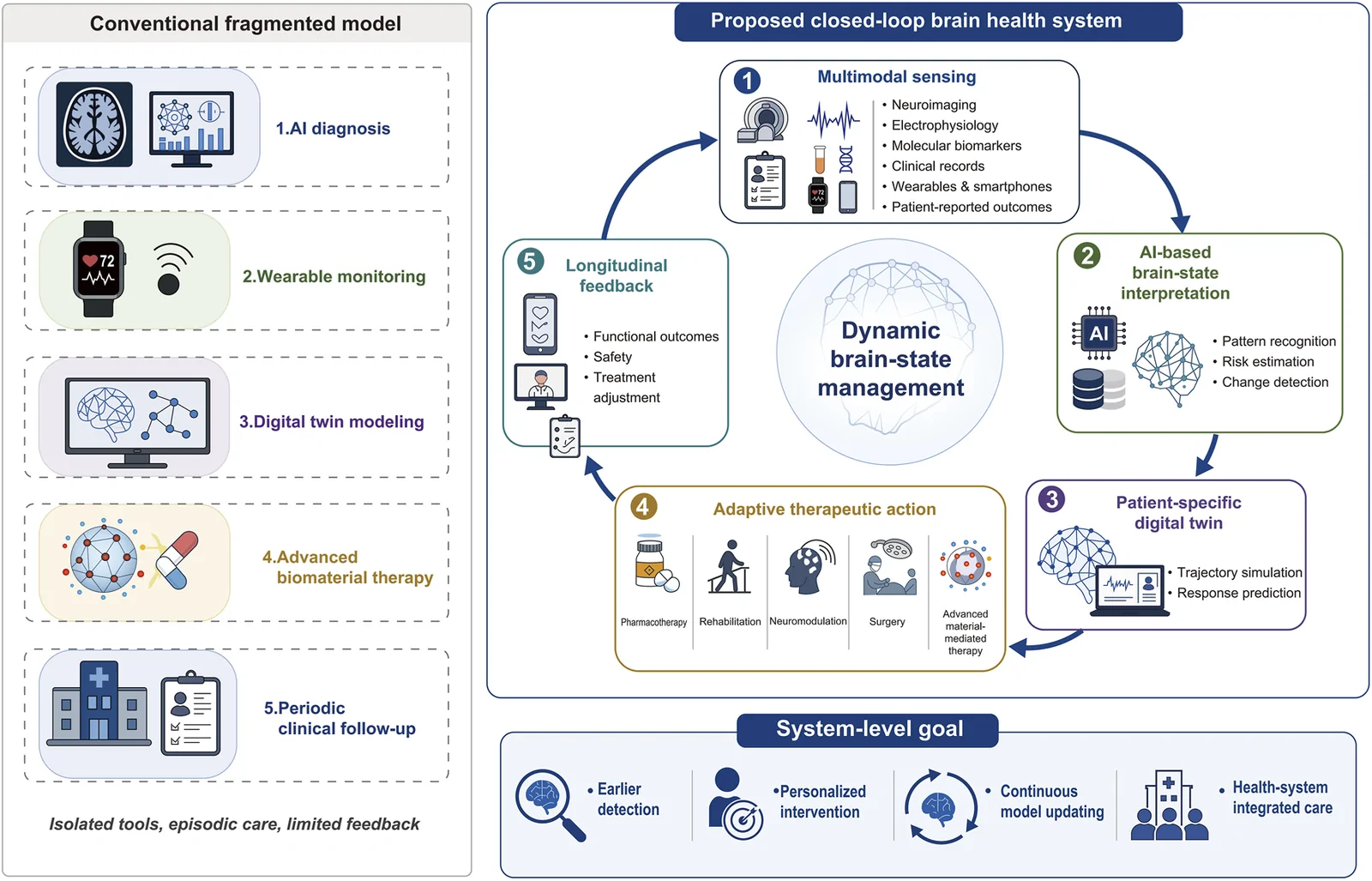
Closed-loop framework for AI-, digital health-, digital twin-, and advanced material-enabled brain health systems. Conventional brain disease innovation often develops artificial intelligence diagnosis, wearable monitoring, digital twin modeling, and advanced biomaterial therapy as separate technological tracks. Although each track can produce valuable advances, isolated development may limit clinical translation. The proposed closed-loop framework begins with multimodal sensing, including neuroimaging, electrophysiology, molecular biomarkers, clinical records, wearable data, smartphone-based digital biomarkers, and patient-reported outcomes. These data are integrated by artificial intelligence models to interpret dynamic brain states and estimate risk. A patient-specific digital twin then simulates disease trajectory and potential intervention effects. Therapeutic action may include pharmacological therapy, rehabilitation, neuromodulation, surgery, behavioral intervention, or advanced material-mediated drug delivery and neural repair. Longitudinal feedback from digital health platforms and clinical follow-up updates the model over time. This framework shifts the evaluation goal from isolated technological feasibility toward adaptive, patient-specific, and health-system-integrated brain disease management.

We propose a closed-loop framework for brain health system innovation built around five connected components. The first component is multimodal sensing, including neuroimaging, electrophysiology, clinical records, molecular biomarkers, wearable data, smartphone signals, environmental measures, and patient-reported outcomes. The second component is artificial intelligence-based brain-state interpretation, in which models identify disease patterns, estimate risk, detect meaningful changes, and support clinical reasoning. The third component is digital twin modeling, in which patient-specific trajectories and intervention scenarios are simulated. The fourth component is adaptive therapeutic action, including pharmacological treatment, rehabilitation, neuromodulation, surgery, behavioral intervention, and advanced material-mediated therapy. The fifth component is longitudinal feedback, in which treatment effects are monitored and used to update the model over time. The relationships among these components and their integration into a continuous adaptive cycle are illustrated in Figure 1. The differences between conventional fragmented brain disease management and the proposed closed-loop system are summarized in Supplementary Table 1.

This framework differs from conventional pipelines in several ways. First, it is circular rather than linear. Traditional care often moves from diagnosis to treatment to follow-up, with limited feedback between stages (Boyd et al., 2022). A closed-loop system continuously updates the patient model. Second, it is patient-specific rather than population-average. Guidelines remain important, but digital and computational tools allow treatment to be adapted to individual trajectories. Third, it is intervention-oriented rather than prediction-only. The purpose of modeling is not merely to forecast decline, but to identify actionable opportunities for changing outcomes. Fourth, it is system-level rather than device-level. The value of a tool depends on how it contributes to the larger care pathway.

For brain disorders, this closed-loop logic is particularly appropriate because disease states are dynamic and interventions often require adjustment. In epilepsy, seizure risk may fluctuate and therapy may need to adapt. In stroke, rehabilitation intensity and strategy should evolve with recovery (Kimberley and Plow, 2025). In neurodegeneration, decline may accelerate or stabilize depending on comorbidity, treatment, and lifestyle factors (Tablante et al., 2025). In brain tumors, surveillance and treatment must respond to changing imaging and molecular information (Zaccagna et al., 2021). A closed-loop system can help align monitoring and intervention with these temporal patterns.

Importantly, this framework does not imply that all components must be fully mature before clinical use. Existing and near-clinical closed-loop systems already provide useful reference points. For example, responsive neurostimulation for epilepsy can detect abnormal neural activity and deliver stimulation in response to patient-specific electrographic patterns (Heck et al., 2014), while hybrid closed-loop insulin delivery systems integrate continuous glucose monitoring, algorithm-based interpretation, and automated insulin adjustment (Brown et al., 2019). Although these examples differ from the broader brain health systems proposed here, they demonstrate that closed-loop medical architectures can move from concept to clinical implementation when sensing, computation, intervention, and feedback are connected. Different brain disorders may require different levels of system complexity. Some applications may begin with AI-supported imaging and wearable follow-up. Others may include digital twin simulation or biomaterial-based intervention. The key point is conceptual: technologies should be designed with integration in mind from the beginning.

### 7.2 Translational readiness should be measured at the system level

A major weakness in current neurotechnology development is that translational readiness is often evaluated at the level of individual performance metrics (Chen and Cherkassky, 2020). An artificial intelligence model may report high classification accuracy. A wearable sensor may show strong signal reliability. A biomaterial may demonstrate effective drug release. A digital twin may simulate disease progression (Sriramulu and Rajendran, 2026). These achievements are important, but none guarantees clinical impact. The system may still fail if it is not interpretable, scalable, affordable, safe, equitable, or compatible with clinical workflow.

We therefore argue that translational readiness should be measured at the system level. For artificial intelligence tools, this means evaluating generalizability across populations, interpretability for clinicians, prospective performance, bias, regulatory compliance, and effect on decision-making. For digital health platforms, it means evaluating adherence, usability, data governance, clinical utility, integration with electronic health records, and burden on clinicians and patients (Rajendran and Selvan, 2025). For biomaterials, it means evaluating manufacturing reproducibility, sterility, long-term safety, delivery feasibility, monitoring capacity, and compatibility with standard neurological care. For digital twins, it means evaluating whether simulation improves decisions rather than merely producing plausible models.

This system-level perspective also affects study design. Retrospective algorithm development is not enough. Small-animal biomaterial studies are not enough. Device validation in healthy volunteers is not enough. The field needs prospective, longitudinal, multidisciplinary, and clinically embedded studies that test whether integrated systems improve outcomes. Outcomes should include not only technical accuracy but also time to diagnosis, functional recovery, adverse events, treatment adjustment, patient quality of life, caregiver burden, clinician workload, cost-effectiveness, and health equity.

Such evaluation is difficult but necessary. Brain disease care is already complex, and poorly integrated technologies may increase complexity rather than reduce it (Zhang R. et al., 2025). A closed-loop system should make care more precise, not more fragmented (Ling et al., 2026). It should help clinicians make better decisions, not replace clinical judgment with opaque outputs. It should empower patients and caregivers, not convert daily life into constant surveillance. It should improve access and outcomes, not widen disparities between technology-rich and technology-poor settings.

### 7.3 Ethical and governance challenges are central, not secondary

The integration of artificial intelligence, digital health, digital twins, and advanced materials raises ethical and governance challenges that are particularly sensitive in brain disease. Brain data can reveal cognitive ability, emotional state, behavior, sleep, movement, social activity, and functional independence. Patients with dementia, severe neurological disability, psychiatric comorbidity, or brain injury may have impaired decision-making capacity (Nainwal et al., 2025). Continuous monitoring can blur the boundary between healthcare and surveillance. Algorithmic predictions may affect access to treatment, insurance, employment, or social support if misused (Montalbano, 2025).

These issues cannot be treated as afterthoughts. Ethical design should begin at the earliest stage of technology development. Patients and caregivers should be involved in defining acceptable monitoring, meaningful outcomes, and tolerable burdens (Hanggodo et al., 2026). Consent processes should account for cognitive impairment and changing capacity over time. Data systems should minimize unnecessary collection, protect privacy, and clarify who can access information. Algorithms should be audited for bias and validated across demographic, socioeconomic, and geographic groups. Clinicians should understand the limits of model outputs and retain responsibility for contextual judgment.

Cybersecurity should also be treated as a direct patient-safety requirement rather than only a data-protection issue. In a closed-loop brain health system, malicious or accidental interference with sensor inputs, data streams, software updates, device communication, or implantable and wearable device control could lead to inappropriate stimulation, drug delivery, rehabilitation adjustment, or risk alerts. Therefore, cybersecurity should be built into the system architecture through secure authentication, encrypted communication, access control, anomaly detection, audit trails, software update management, fail-safe modes, and human override mechanisms. These safeguards are particularly important when digital outputs are linked to therapeutic actions that may affect neural activity, medication exposure, or patient behavior (Cybersecurity in Medical Devices, 2026).

Advanced materials introduce additional governance concerns. Brain-targeted or implanted systems may have long-term effects that are difficult to reverse. Stimuli-responsive materials, neural interfaces, and regenerative constructs may interact with brain function in complex ways. Regulatory frameworks must therefore address not only material safety but also monitoring, controllability, and post-market follow-up. When material-based interventions are linked to digital systems or artificial intelligence-guided decisions, accountability becomes even more complex.

Regulatory evaluation may be especially challenging when closed-loop brain health systems combine AI software, wearable sensors, implantable hardware, and advanced biomaterials within a single adaptive care architecture. Conventional approval pathways are often designed for products with relatively stable functions and clearly defined boundaries, whereas closed-loop systems may change over time as algorithms are updated, patient data accumulate, device performance evolves, and therapeutic responses feed back into the system (FDA, 2025). Future regulatory strategies may therefore need to consider system boundaries, component-level risk classification, interoperability, cybersecurity, algorithm change management, real-world performance monitoring, post-deployment surveillance, and clear responsibility for human oversight and clinical decision-making. These requirements are not secondary to translation; they are part of whether an integrated brain health system can be safely and responsibly deployed.

We suggest that governance should be built into closed-loop brain health systems as an explicit design layer. This includes technical standards, clinical validation pathways, data protection, algorithm transparency, patient engagement, regulatory science, and post-deployment surveillance. Without such governance, even technically impressive systems may fail to earn trust or achieve clinical adoption.

## 8 Discussion

### 8.1 Translational promise and unresolved barriers

The convergence of artificial intelligence, digital health, digital twins, and advanced materials has created a genuine opportunity to transform brain disease care. These technologies address complementary limitations of current neurology. Artificial intelligence can interpret complex data. Digital health can monitor patients beyond the clinic. Digital twins can simulate disease trajectories and intervention effects. Advanced materials can deliver or modulate therapy in ways that conventional drugs often cannot. Together, they could support a transition from reactive, episodic care to proactive, adaptive, and patient-specific brain health management.

However, this translational promise should be interpreted cautiously. Many artificial intelligence models still lack external validation, prospective testing, clinical interpretability, and evidence of outcome improvement. Many digital biomarkers remain technically interesting but clinically underdefined. Digital twins are conceptually powerful but often immature in biological realism and clinical deployment. Many biomaterial platforms show promise in preclinical settings but face major barriers in manufacturing, safety, delivery, and regulatory approval. Most importantly, these technologies are often developed separately, which limits their ability to change care pathways.

The central barrier is therefore not only technical but architectural. The field does not simply need better algorithms, more sensors, more complex models, or more sophisticated materials. It needs a coherent translational architecture that defines how these components work together. Without such architecture, brain disease innovation may produce many impressive tools but few durable improvements in clinical outcomes.

### 8.2 Future progress will depend on redefining what counts as success

Our central argument is straightforward: brain disease innovation should stop asking only whether individual technologies can perform isolated tasks, and start asking whether they can create adaptive systems that improve brain health over time. This shift has practical consequences.

First, it changes what should be measured. Studies should evaluate not only diagnostic accuracy, sensor reliability, simulation plausibility, or drug release profiles, but also longitudinal disease modeling, treatment adaptation, functional outcomes, patient experience, workflow integration, and health-system impact. Second, it changes what should be optimized. Instead of maximizing single-domain performance, investigators should optimize compatibility among data streams, models, materials, clinicians, patients, and care settings. Third, it changes what should count as translational readiness. Technologies should be judged not only by feasibility but also by reproducibility, interpretability, scalability, safety, equity, and ability to support real clinical decisions.

In our view, future progress depends on three converging priorities. The first is brain-state modeling: developing artificial intelligence and digital twin systems that represent disease as a dynamic, patient-specific process rather than a static label. The second is programmable intervention: designing biomaterials, neurotechnologies, and therapeutic strategies that can respond to disease states and be monitored over time. The third is health-system integration: building digital platforms, governance structures, validation pathways, and clinical workflows that allow these technologies to function together.

If these priorities are pursued together, brain disease care may move toward closed-loop precision neurology. If they are pursued separately, the field may continue to generate fragmented innovations that remain difficult to translate. The next major advances, in our view, will come from approaches that integrate artificial intelligence, digital health, digital twin modeling, and advanced materials as components of a single adaptive brain health system.

## Data Availability

The original contributions presented in the study are included in the article/Supplementary Material, further inquiries can be directed to the corresponding author.

## References

[B1] Akwa Y. (2020). Steroids and lzheimer’s disease: changes associated with pathology and therapeutic potential. Int. J. Mol. Sci. 21, 4812. 10.3390/ijms21134812 32646017 PMC7370115

[B2] Aliotta E. Jeong J. Paudyal R. Grkovski M. Diplas B. Han J. (2025). Predicting and monitoring response to head and neck cancer radiotherapy using multimodality imaging and radiobiological digital twin simulations. Phys. Med. Biol. 70, 115016. 10.1088/1361-6560/add9de PMC12169438 40378867

[B3] Almarghalani D. A. Sha X. Mrak R. E. Shah Z. A. (2023). Spatiotemporal cofilin signaling, microglial activation, neuroinflammation, and cognitive impairment following hemorrhagic brain injury. Cells 12, 1153. 10.3390/cells12081153 37190062 PMC10137307

[B4] Altuna M. García-Sebastián M. Cipriani R. Capetillo-Zarate E. Alberdi E. Estanga A. (2025). Stepwise approach to alzheimer’s disease diagnosis in primary care using cognitive screening, risk factors, neuroimaging and plasma biomarkers. Sci. Rep. 15, 31526. 10.1038/s41598-025-17394-3 40866545 PMC12391538

[B5] Alves P. A. Camargo L. C. de Souza G. M. Mortari M. R. Homem-de-Mello M. (2025). Computational modeling of pharmaceuticals with an emphasis on crossing the blood-brain barrier. Pharmaceuticals 18, 217. 10.3390/ph18020217 40006031 PMC11860133

[B6] Anderson J. M. Rodriguez A. Chang D. T. (2008). Foreign body reaction to biomaterials. Seminars Immunol. 20, 86–100. 10.1016/j.smim.2007.11.004 18162407 PMC2327202

[B7] Antar T. Morris H. R. Faghri F. Leonard H. L. Nalls M. A. Singleton A. B. (2021). Longitudinal risk factors for developing depressive symptoms in Parkinson’s disease. J. Neurological Sciences 429, 117615. 10.1016/j.jns.2021.117615 PMC8653575 34492572

[B8] Aswendt M. Pallast N. Wieters F. Baues M. Hoehn M. Fink G. R. (2021). Lesion Size- and location-dependent recruitment of contralesional thalamus and motor cortex facilitates recovery after stroke in mice. Transl. Stroke Res. 12, 87–97. 10.1007/s12975-020-00802-3 32166716 PMC7803721

[B9] Awasthi A. Vu A. M. Le N. Deng Z. Maulik S. Agrawal R. (2025). Modeling radiologists’ cognitive processes using a digital gaze twin to enhance radiology training. Sci. Rep. 15, 13685. 10.1038/s41598-025-97935-y 40258898 PMC12012060

[B10] Aziz S. Ali A. A. M. Aslam H. ul Ain N. Tariq A. Sohail Z. (2025). Wearable artificial intelligence for epilepsy: scoping review. J. Med. Internet Res. 27, e73593. 10.2196/73593 41172347 PMC12578435

[B11] Bachmann T. Schroeter M. L. Chen K. Reiman E. M. Weise C. M. (2023). Longitudinal changes in surface based brain morphometry measures in amnestic mild cognitive impairment and Alzheimer’s disease. NeuroImage Clin. 38, 103371. 10.1016/j.nicl.2023.103371 36924681 PMC10025277

[B12] Bahado-Singh R. O. Vishweswaraiah S. Turkoglu O. Graham S. F. Radhakrishna U. (2023). Alzheimer’s precision neurology: epigenetics of cytochrome P450 genes in circulating cell-free DNA for disease prediction and mechanism. Int. J. Mol. Sci. 24, 2876. 10.3390/ijms24032876 36769199 PMC9917756

[B13] Banerjee P. Koumans H. Weech M. D. Wilson M. Rivera-Morales M. Ganti L. (2021). AHORA: a Spanish language tool to identify acute stroke symptoms. Stroke Vasc. Neurology 7, 176–178. 10.1136/svn-2021-001280 PMC9067269 34702749

[B167] Bhasha S. J. Panda S. (2024). Data privacy preservation and authentication scheme for secured IoMT communication using enhanced heuristic approach with deep learning. Cybern. Syst. 1–42. 10.1080/01969722.2024.2343982

[B14] Bier V. Borgonovo E. Cox T. Rudin C. (2025). Artificial intelligence for risk analysis and the risks of artificial intelligence: part 1. Risk Analysis An Official Publ. Soc. Risk Analysis 45, 735–737. 10.1111/risa.70017 40079462

[B15] Bosak N. Branco P. Kuperman P. Buxbaum C. Cohen R. M. Fadel S. (2022). Brain connectivity predicts chronic pain in acute mild traumatic brain injury. Ann. Neurology 92, 819–833. 10.1002/ana.26463 PMC9826527 36082761

[B16] Boyd N. D. Naasan G. Harrison K. L. Garrett S. B. D'Aguiar Rosa T. Pérez‐Cerpa B. (2022). Characteristics of people with dementia lost to follow-up from a dementia care center. Int. Journal Geriatric Psychiatry 37, gps.5628. 10.1002/gps.5628 PMC8740544 34590336

[B17] Boyle A. J. Gaudet V. C. Black S. E. Vasdev N. Rosa-Neto P. Zukotynski K. A. (2021). Artificial intelligence for molecular neuroimaging. Ann. Transl. Med. 9, 822. 10.21037/atm-20-6220 34268435 PMC8246223

[B18] Brown S. A. Kovatchev B. P. Raghinaru D. Lum J. W. Buckingham B. A. Kudva Y. C. (2019). Six-month randomized, multicenter trial of closed-loop control in type 1 diabetes. N. Engl. J. Med. 381, 1707–1717. 10.1056/nejmoa1907863 31618560 PMC7076915

[B19] Busia P. Leone G. Matticola A. Raffo L. Meloni P. (2025). Wearable epilepsy seizure detection on FPGA with spiking neural networks. IEEE Transactions Biomedical Circuits Systems 19, 1175–1186. 10.1109/tbcas.2025.3575327 40445816

[B20] Campagnini S. Arienti C. Patrini M. Liuzzi P. Mannini A. Carrozza M. C. (2022). Machine learning methods for functional recovery prediction and prognosis in post-stroke rehabilitation: a systematic review. J. NeuroEngineering Rehabilitation 19, 54. 10.1186/s12984-022-01032-4 PMC9166382 35659246

[B21] Chen H.-H. Cherkassky V. (2020). Performance metrics for online seizure prediction. Neural Networks The Official Journal Int. Neural Netw. Soc. 128, 22–32. 10.1016/j.neunet.2020.04.022 32387921 PMC7340210

[B22] Coates L. Shi J. Rochester L. Del Din S. Pantall A. (2020). Entropy of real-world gait in parkinson’s disease determined from wearable sensors as a digital marker of altered ambulatory behavior. Sensors Basel, Switz. 20, 2631. 10.3390/s20092631 32380692 PMC7249239

[B23] Cornelison C. Fadel S. (2022). Clickable biomaterials for modulating neuroinflammation. Int. J. Mol. Sci. 23, 8496. 10.3390/ijms23158496 35955631 PMC9369181

[B24] Couch C. Mallah K. Borucki D. M. Bonilha H. S. Tomlinson S. (2022). State of the science in inflammation and stroke recovery: a systematic review. Ann. Physical Rehabilitation Medicine 65, 101546. 10.1016/j.rehab.2021.101546 PMC9018463 34098132

[B25] Cowie M. R. Lam C. S. P. (2021). Remote monitoring and digital health tools in CVD management. Nat. Rev. Cardiol. 18, 457–458. 10.1038/s41569-021-00548-x 33824486 PMC8023506

[B26] Cybersecurity in Medical Devices (2026). Quality Management System Considerations and Content of Premarket Submissions.

[B27] Daneault J.-F. Vergara-Diaz G. Parisi F. Admati C. Alfonso C. Bertoli M. (2021). Accelerometer data collected with a minimum set of wearable sensors from subjects with Parkinson’s disease. Sci. Data 8, 48. 10.1038/s41597-021-00830-0 33547309 PMC7865022

[B28] Dell K. L. Payne D. E. Kremen V. Maturana M. I. Gerla V. Nejedly P. (2021). Seizure likelihood varies with day-to-day variations in sleep duration in patients with refractory focal epilepsy: a longitudinal electroencephalography investigation. EClinicalMedicine 37, 100934. 10.1016/j.eclinm.2021.100934 34386736 PMC8343264

[B29] Diana-Albelda C. García-Martín Á. Bescos J. (2025). A review on deep learning methods for glioma segmentation, limitations, and future perspectives. J. Imaging 11, 269. 10.3390/jimaging11080269 40863479 PMC12387613

[B30] Digital Twin (2025). A digital twin that interprets and refines chemical mechanisms, Nat. Comput. Sci. 5 713–714. 40890530 10.1038/s43588-025-00859-w

[B31] Etholén A. Roos T. Hänninen M. Bouri I. Kulmala J. Rahkonen O. (2025). Forecasting subjective cognitive decline: AI approach using dynamic bayesian networks. J. Med. Internet Res. 27, e65028. 10.2196/65028 40327854 PMC12093071

[B32] Eyal S. (2025). Commentary: therapeutic seizure monitoring: a closed-loop device for direct delivery of antiseizure therapeutics. Epilepsy Curr. 25, 190–191. 10.1177/15357597251320190 40161510 PMC11949755

[B33] Fedotchev A. Parin S. Polevaya S. Zemlianaia A. (2021). Human body rhythms in the development of non-invasive methods of closed-loop adaptive neurostimulation. J. Personalized Med. 11, 437. 10.3390/jpm11050437 PMC8161182 34065196

[B34] Felius R. A. W. Punt M. Wouda N. C. Geerars M. Bruijn S. M. Wittink H. (2025). Mapping trajectories of gait recovery in clinical stroke rehabilitation. Neurorehabilitation Neural Repair 39, 274–285. 10.1177/15459683241304350 39810283

[B35] FDA (2025). Marketing Submission Recommendations for a Predetermined Change Control Plan for Artificial Intelligence-Enabled Device Software Functions.

[B36] Gao X. Chen X. Lin M. Yue W. Hu H. Qin S. (2024). A wearable echomyography system based on a single transducer. Nat. Electron. 7, 1035–1046. 10.1038/s41928-024-01271-4 40677283 PMC12269893

[B37] Gao P. Li X. Du X. Liu S. Xu Y. (2021). Diagnostic and therapeutic potential of exosomes in neurodegenerative diseases. Front. Aging Neurosci. 13, 790863. 10.3389/fnagi.2021.790863 34975460 PMC8717921

[B38] Gao X. Wang X. Labelle-Dumais C. Gould D. B. Chaumeil M. M. (2025). Multimodal neuroimaging of Col4a1-mutant mouse models of Gould syndrome. Front. Neurosci. 19, 1639871. 10.3389/fnins.2025.1639871 41311701 PMC12647094

[B39] Garcia S. F. Gray R. J. Sparano J. A. Tevaarwerk A. J. Carlos R. C. Yanez B. (2022). Fatigue and endocrine symptoms among women with early breast cancer randomized to endocrine *versus* chemoendocrine therapy: results from the TAILORx patient-reported outcomes substudy. Cancer 128, 536–546. 10.1002/cncr.33939 34614209 PMC8776586

[B40] Germanotta M. Iacovelli C. Aprile I. (2022). Evaluation of gait smoothness in patients with stroke undergoing rehabilitation: Comparison between two metrics. Int. J. Environ. Res. Public Health 19, 13440. 10.3390/ijerph192013440 36294017 PMC9603299

[B41] Ghafarfaraji S. (2025). AI-based recognition of facial and micro-expressions for the diagnosis of mental and neurological disorders: a systematic review. BMC Psychiatry 26, 78. 10.1186/s12888-025-07739-7 41449384 PMC12849402

[B42] Ghezzi L. Kosa P. Greenwood M. Alvarez E. Freedman C. L. Cross A. H. (2026). From diagnosis to disease staging: multisite validation of cerebrospinal fluid molecular tests in multiple sclerosis. Ann. Neurology 99, 328–340. 10.1002/ana.78047 PMC12894485 41041831

[B43] Ghosn N. J. Xie K. Pattnaik A. R. Gugger J. J. Ellis C. A. Sweeney E. (2023). A pharmacokinetic model of antiseizure medication load to guide care in the epilepsy monitoring unit. Epilepsia 64, 1236–1247. 10.1111/epi.17558 36815252 PMC10424095

[B44] Graf S. Hofer G. Hirschmann R. Lehmann R. Cavelti-Weder C. (2025). Real-world evidence supporting the use of advanced hybrid closed loop in poorly controlled type 1 diabetes patients. Diabetes Res. Clin. Pract. 222 (2025), 112035. 10.1016/j.diabres.2025.112035 39929337

[B45] Gu Y. Zhao P. Feng W. Xia X. Tian X. Yan Y. (2022). Structural brain network measures in elderly patients with cerebral small vessel disease and depressive symptoms. BMC Geriatr. 22, 568. 10.1186/s12877-022-03245-7 35810313 PMC9270825

[B46] Gugger J. J. Sinha N. Huang Y. Walter A. E. Lynch C. Kalyani P. (2023). Structural brain network deviations predict recovery after traumatic brain injury. NeuroImage Clin. 38, 103392. 10.1016/j.nicl.2023.103392 37018913 PMC10122019

[B47] Haaksma M. L. Vilela L. R. Marengoni A. Calderón-Larrañaga A. Leoutsakos J. M. S. Olde Rikkert M. G. M. (2017). Comorbidity and progression of late onset Alzheimer’s disease: a systematic review. PloS One 12, e0177044. 10.1371/journal.pone.0177044 28472200 PMC5417646

[B48] Hadjichrysanthou C. Evans S. Bajaj S. Siakallis L. C. McRae-McKee K. de Wolf F. (2020). The dynamics of biomarkers across the clinical spectrum of Alzheimer’s disease. Alzheimer’s Res. and Ther. 12, 74. 10.1186/s13195-020-00636-z 32534594 PMC7293779

[B49] Han K. Joung J. F. Han M. Sung W. Kang Y. n. (2022). Locoregional recurrence prediction using a deep neural network of radiological and radiotherapy images. J. Personalized Med. 12, 143. 10.3390/jpm12020143 PMC8875706 35207631

[B50] Hanggodo S. Koustov T. Sharp K. Grant-Kels J. M. (2026). Ethical implications of artificial intelligence-written personal statements in dermatology. J. Am. Acad. Dermatology 94, e5–e6. 10.1016/j.jaad.2025.03.030 40107505

[B51] Hays S. A. Adehunoluwa E. A. Epperson J. D. Malley K. M. Porter A. L. Gallaway H. L. (2026). Closed-loop vagus nerve stimulation delivered with a miniaturized system produces lasting recovery in individuals with chronic stroke. Stroke 57, 38–49. 10.1161/strokeaha.125.052937 41054846 PMC12614473

[B52] Heck C. N. King-Stephens D. Massey A. D. Nair D. R. Jobst B. C. Barkley G. L. (2014). Two-year seizure reduction in adults with medically intractable partial onset epilepsy treated with responsive neurostimulation: final results of the RNS system pivotal trial. Epilepsia 55, 432–441. 10.1111/epi.12534 24621228 PMC4233950

[B53] Hope T. M. H. Nardo D. Holland R. Ondobaka S. Akkad H. Price C. J. (2021). Lesion site and therapy time predict responses to a therapy for anomia after stroke: a prognostic model development study. Sci. Rep. 11, 18572. 10.1038/s41598-021-97916-x 34535718 PMC8448867

[B54] Hu X. Ricci S. Naranjo S. Hill Z. Gawason P. (2021). Protein and polysaccharide-based electroactive and conductive materials for biomedical applications. Molecules 26, 4499. 10.3390/molecules26154499 34361653 PMC8348981

[B55] Jeppesen J. Fuglsang-Frederiksen A. Johansen P. Christensen J. Wüstenhagen S. Tankisi H. (2020). Seizure detection using heart rate variability: a prospective validation study. Epilepsia 61 (Suppl. 1), S41–S46. 10.1111/epi.16511 32378197

[B56] Johansson B. (2021). Mental fatigue after mild traumatic brain injury in relation to cognitive tests and brain imaging methods. Int. J. Environ. Res. Public Health 18, 5955. 10.3390/ijerph18115955 34199339 PMC8199529

[B57] Keser Z. Buchl S. C. Seven N. A. Markota M. Clark H. M. Jones D. T. (2022). Electroencephalogram (EEG) with or without transcranial Magnetic stimulation (TMS) as biomarkers for post-stroke recovery: a narrative review. Front. Neurology 13, 827866. 10.3389/fneur.2022.827866 PMC8902309 35273559

[B58] Khan A. Shim V. Fernandez J. Kasabov N. K. Wang A. (2025). Review of deep learning models with spiking neural networks for modeling and analysis of multimodal neuroimaging data. Front. Neurosci. 19, 1623497. 10.3389/fnins.2025.1623497 41322350 PMC12660199

[B59] Kim D. O’Shea L. M. Aghamohammadi N. R. (2025). Insights into the dependence of post-stroke motor recovery on the initial corticospinal tract connectivity from a computational model. J. Neuroengineering Rehabilitation 22, 8. 10.1186/s12984-024-01513-8 PMC11749208 39833900

[B60] Kimberley T. J. Plow E. B. (2025). Rehabilitation drives post-stroke motor recovery. Lancet. Neurology 24, 373–375. 10.1016/s1474-4422(25)00100-0 40157381

[B61] Koros C. Stanitsa E. Angelopoulou E. Papageorgiou S. G. Stefanis L. (2025). Cognitive decline in parkinsonism: from clinical phenotypes to the genetic background. Biomedicines 13, 1624. 10.3390/biomedicines13071624 40722695 PMC12292138

[B62] Kouwenberg V. van Santwijk L. Meijer F. J. A. Henssen D. (2022). Reliability of dynamic susceptibility contrast perfusion metrics in pre- and post-treatment glioma. Cancer Imaging 22, 28. 10.1186/s40644-022-00466-2 35715866 PMC9205029

[B63] Lea-Henry T. N. Carland J. E. Stocker S. L. Sevastos J. Roberts D. M. (2018). Clinical pharmacokinetics in kidney disease. Clin. J. Am. Soc. Nephrol. CJASN 13, 1085–1095. 10.2215/CJN.00340118 29934432 PMC6032582

[B64] Lee W. J. Yoon C. W. Kim S.-W. Jeong H. J. Seo S. Na D. L. (2021). Effects of Alzheimer’s and vascular pathologies on structural connectivity in Early- and late-onset Alzheimer’s disease. Front. Neurosci. 15, 606600. 10.3389/fnins.2021.606600 33664644 PMC7921324

[B65] Lee H. Cho M. Lee S. W. Park S. (2025). Predicting sleep quality with digital biomarkers and artificial neural networks. Front. Psychiatry 16, 1591448. 10.3389/fpsyt.2025.1591448 40740269 PMC12308496

[B66] Li D. Patel C. B. Xu G. Iagaru A. Zhu Z. Zhang L. (2020). Visualization of diagnostic and therapeutic targets in Glioma with molecular imaging. Front. Immunol. 11, 592389. 10.3389/fimmu.2020.592389 33193439 PMC7662122

[B67] Li Q. Wang W. Guo Q. Jiang L. Qiao K. Hu Y. (2025). Toward neuroimaging-based diagnostic support: a deep learning approach with a closed-loop system for psychiatric classification. iScience 28, 114100. 10.1016/j.isci.2025.114100 41399512 PMC12702231

[B68] Ling X. Zhu Y. Li M. Xie Z. Cao L. Song W. (2026). A closed-loop hybrid discovery System of type I photosensitizers for hypoxic tumor therapy. Adv. Sci. 13, e15103. 10.1002/advs.202515103 PMC12903996 41386764

[B69] Liptzin D. R. Pickett K. Brinton J. T. Agarwal A. Fishman M. P. Casey A. (2020). Neuroendocrine cell hyperplasia of infancy. Clinical score and comorbidities. Ann. Am. Thorac. Soc. 17, 724–728. 10.1513/AnnalsATS.201908-617OC 32109152 PMC7258416

[B70] Liu S.-B. Wang J.-H. Yuan R.-Y. Zhao W. X. Li L. Wang Q. H. (2020). Real-time and ultrahigh accuracy image synthesis algorithm for full field of view imaging system. Sci. Rep. 10, 12389. 10.1038/s41598-020-69353-9 32709880 PMC7382481

[B71] Liu M. Qiao Y. Cheng S. Su Z. Li Z. Gao P. (2025). 3D bioprinted microneedle patches loaded with gambogic acid-iron-doxorubicin nanozymes for postoperative glioma *in situ* therapy *via* ferroptosis synergistic chemosensitization. J. Nanobiotechnol. 23, 755. 10.1186/s12951-025-03790-4 PMC12676811 41272423

[B72] Lopes T. Reis Carneiro M. Morgadinho A. Reis Carneiro D. Tavakoli M. (2025). ParCuR-A novel AI-Enabled gait cueing wearable for patients with parkinson’s disease. Sensors 25, 7077. 10.3390/s25227077 41305286 PMC12656022

[B73] Lotz B. Bothe F. Deubel A.-K. Hesse E. Renz Y. Werner C. (2021). Preclinical testing of new hydrogel materials for cartilage repair: overcoming fixation issues in a large animal model. Int. J. Biomater. 2021, 5583815–14. 10.1155/2021/5583815 34239571 PMC8235960

[B74] Lu Y. Wang L. Murai T. Wu J. Liang D. Zhang Z. (2025). Detection of structural-functional coupling abnormalities using multimodal brain networks in Alzheimer’s disease: a comparison of three computational models. NeuroImage. Clin. 46, 103764. 10.1016/j.nicl.2025.103764 40101672 PMC11960660

[B75] Ma Y. Lai P. Sha Z. Li B. Wu J. Zhou X. (2025). TME-responsive nanocomposite hydrogel with targeted capacity for enhanced synergistic chemoimmunotherapy of MYC-amplified osteosarcoma. Bioact. Mater. 47, 83–99. 10.1016/j.bioactmat.2025.01.006 39897587 PMC11783017

[B76] Maadi M. Akbarzadeh Khorshidi H. Aickelin U. (2021). A review on Human–AI interaction in machine learning and insights for medical applications. Int. J. Environ. Res. Public Health 18, 2121. 10.3390/ijerph18042121 33671609 PMC7926732

[B77] Mancini M. Afshari M. Almeida Q. Amundsen-Huffmaster S. Balfany K. Camicioli R. (2025). Digital gait biomarkers in Parkinson’s disease: susceptibility/risk, progression, response to exercise, and prognosis. NPJ Parkinson’s Disease 11, 51. 10.1038/s41531-025-00897-1 PMC11928532 40118834

[B78] Mata-Martínez E. Díaz-Muñoz M. Vázquez-Cuevas F. G. (2022). Glial cells and brain diseases: inflammasomes as relevant pathological entities. Front. Cell. Neurosci. 16, 929529. 10.3389/fncel.2022.929529 35783102 PMC9243488

[B79] Matejuk A. Benedek G. Bucala R. Matejuk S. Offner H. Vandenbark A. A. (2024). MIF contribution to progressive brain diseases. J. Neuroinflammation 21, 8. 10.1186/s12974-023-02993-6 38178143 PMC10765708

[B80] Mathieu C. Sims E. K. Chatenoud L. James E. A. Atkinson M. A. Herold K. C. (2026). Toward disease-modifying therapies in type 1 diabetes: focus on Teplizumab. Diabetes Care 49, 365–374. 10.2337/dci25-0066 41196630 PMC12925991

[B81] McGrath E. R. Beiser A. S. O’Donnell A. Himali J. J. Pase M. P. Satizabal C. L. (2022). Determining vascular risk factors for dementia and dementia risk prediction across Mid-to later life. Neurology 99, e142–e153. 10.1212/wnl.0000000000200521 35584926 PMC9280997

[B82] Meyer A. N. D. Giardina T. D. Spitzmueller C. Shahid U. Scott T. M. T. Singh H. (2020). Patient perspectives on the usefulness of an artificial Intelligence-assisted symptom checker: cross-sectional survey study. J. Med. Internet Res. 22, e14679. 10.2196/14679 32012052 PMC7055765

[B83] Miller D. J. Sargent C. Roach G. D. (2022). A validation of six wearable devices for estimating sleep, heart rate and heart rate variability in healthy adults. Sensors Basel, Switz. 22, 6317. 10.3390/s22166317 PMC9412437 36016077

[B84] Mohan D. M. Khandoker A. H. Wasti S. A. Ismail Ibrahim Ismail Alali S. Jelinek H. F. Khalaf K. (2021). Assessment methods of post-stroke gait: a scoping review of technology-driven approaches to gait characterization and analysis. Front. Neurology 12, 650024. 10.3389/fneur.2021.650024 PMC8217618 34168608

[B85] Montalbano P. (2025). Ethical and legal considerations of medical artificial intelligence. Prim. Care 52, 769–779. 10.1016/j.pop.2025.07.009 41110915

[B86] Moon S. Song H.-J. Sharma V. D. Lyons K. E. Pahwa R. Akinwuntan A. E. (2020). Classification of Parkinson’s disease and essential tremor based on balance and gait characteristics from wearable motion sensors *via* machine learning techniques: a data-driven approach. J. Neuroengineering Rehabilitation 17, 125. 10.1186/s12984-020-00756-5 32917244 PMC7488406

[B87] Mouridsen K. Thurner P. Zaharchuk G. (2020). Artificial intelligence applications in stroke. Stroke 51, 2573–2579. 10.1161/STROKEAHA.119.027479 32693750

[B88] Myers J. Owoc K. Fonda H. Chan K. Oo T. Z. Nallamshetty S. (2025). Impact of home-based cardiac rehabilitation on physical function, outcomes, and costs. J. Cardiopulm. Rehabilitation Prev. 45, 200–206. 10.1097/hcr.0000000000000931 40167501

[B89] Nainwal M. Madhu Sudhana Reddy Y. Dhaygude A. D. Ramesh G. Govindasamy C. Mouli D. V. V. V. C. H. (2025). A novel framework of IoT-based multi-disease monitoring system using heuristic improvement of adaptive dilated and attention-based deep learning approaches. Cybern. Syst. 57 (6), 1084–1129. 10.1080/01969722.2025.2470786

[B90] Nakaoku Y. Ogata S. Nemoto K. Kakuta C. Kiyoshige E. Teramoto K. (2025). Combinations of multimodal neuroimaging biomarkers and cognitive test scores to identify patients with cognitive impairment. Front. Aging Neurosci. 17, 1650629. 10.3389/fnagi.2025.1650629 40881196 PMC12380820

[B91] Narodova E. A. (2025). Digital tools for seizure monitoring and self-management in epilepsy: a narrative review. J. Clin. Med. 14, 8701. 10.3390/jcm14248701 41464603 PMC12733385

[B92] Nevler N. Niehoff D. Gleixner A. M. Dodge S. Vogel A. P. Hill D. L. (2025). Developing digital health technologies for frontotemporal degeneration. Alzheimer’s and Dementia J. Alzheimer’s Assoc. 21, e70082. 10.1002/alz.70082 PMC12032191 40285380

[B93] Nham B. Akdal G. Young A. S. Özçelik P. Tanrıverdizade T. Ala R. T. (2023). Capturing nystagmus in the emergency room: posterior circulation stroke *versus* acute vestibular neuritis. J. Neurology 270, 632–641. 10.1007/s00415-022-11202-y PMC9886594 35849153

[B94] Otgonbaatar C. Kim H. Jeon P.-H. Jeon S. H. Cha S. J. Ryu J. K. (2025). Detection of intracranial hemorrhage using ultralow-dose brain computed tomography with deep learning reconstruction *versus* conventional-dose computed tomography. BMC Medical Imaging 25, 522. 10.1186/s12880-025-02082-5 41286703 PMC12751909

[B95] Otte K. Ellermeyer T. Vater T.-S. Voigt M. Kroneberg D. Rasche L. (2020). Instrumental assessment of stepping in place captures clinically relevant motor symptoms of parkinson’s disease. Sensors Basel, Switz. 20, 5465. 10.3390/s20195465 32977647 PMC7582555

[B96] Pardridge W. M. (2022). Advanced blood-brain barrier drug delivery. Pharmaceutics 15, 93. 10.3390/pharmaceutics15010093 36678722 PMC9866552

[B97] Paul S. N. Wingenfeld K. Otte C. Meijer O. C. (2022). Brain mineralocorticoid receptor in health and disease: from molecular signalling to cognitive and emotional function. Br. J. Pharmacol. 179, 3205–3219. 10.1111/bph.15835 35297038 PMC9323486

[B98] Pearson J. Badenoch J. B. Van Wamelen D. (2025). Using prodromal non-motor symptoms to predict Parkinson’s disease onset and motor phenotype. J. Neurol. Neurosurg. Psychiatry 96, 1215–1221. 10.1136/jnnp-2024-335429 40537250

[B99] Pease M. Gonzalez-Martinez J. Puccio A. Nwachuku E. Castellano J. F. Okonkwo D. O. (2022). Risk factors and incidence of epilepsy after severe traumatic brain injury. Ann. Neurol. 92, 663–669. 10.1002/ana.26443 35713346 PMC9489614

[B100] Peña-Ortega F. Robles-Gómez Á. A. Xolalpa-Cueva L. (2022). Microtubules as regulators of neural network shape and function: focus on excitability, plasticity and memory. Cells 11, 923. 10.3390/cells11060923 35326374 PMC8946818

[B101] Perrigue P. M. Murray R. A. Mielcarek A. Henschke A. Moya S. E. (2021). Degradation of drug delivery nanocarriers and payload release: a review of physical methods for tracing nanocarrier biological fate. Pharmaceutics 13, 770. 10.3390/pharmaceutics13060770 34064155 PMC8224277

[B102] Prenkaj B. Aragona D. Flaborea A. Galasso F. Gravina S. Podo L. (2023). A self-supervised algorithm to detect signs of social isolation in the elderly from daily activity sequences. Artif. Intell. Med. 135, 102454. 10.1016/j.artmed.2022.102454 36628782

[B103] Qiu B. Bessler N. Figler K. Buchholz M. Rios A. C. Malda J. (2020). Bioprinting neural systems to model central nervous system diseases. Adv. Funct. Mater. 30, 1910250. 10.1002/adfm.201910250 34566552 PMC8444304

[B104] Quan G. Zhang K. Liu Y. Ren J. L. Huang D. Wang W. (2021). Role of dynamic susceptibility contrast perfusion MRI in glioma progression evaluation. J. Oncol. 2021, 1696387–1696389. 10.1155/2021/1696387 33628239 PMC7886570

[B105] Rabbito A. Dulewicz M. Kulczyńska-Przybik A. Mroczko B. (2020). Biochemical markers in alzheimer’s disease. Int. J. Mol. Sci. 21, 1989. 10.3390/ijms21061989 32183332 PMC7139967

[B106] Rajendran M. Selvan S. (2025). A novel residual-attention deep learning model for secure multimodal biometric recognition. Cybern. Syst. 0, 1–47. 10.1080/01969722.2025.2566665

[B107] Raspa A. Gelain F. (2021). Mimicking extracellular matrix *via* engineered nanostructured biomaterials for neural repair. Curr. Neuropharmacol. 19, 2110–2124. 10.2174/1570159X18666201111111102 33176654 PMC9185766

[B108] Ren X. Lin J. Stebbins G. T. Goetz C. G. Luo S. (2021). Prognostic modeling of parkinson’s disease progression using early longitudinal patterns of change. Mov. Disorders Official Journal Mov. Disord. Soc. 36, 2853–2861. 10.1002/mds.28730 PMC8688189 34327755

[B109] Rinne J. O. Jucaite A. Cselényi Z. Farde L. (2022). Glia imaging shows clinical utility in differentiating Parkinson’s disease from multiple system atrophy. Mov. Disord. 37, 1776–1778. 10.1002/mds.29078 35666059 PMC9541833

[B110] Rondina J. Nachev P. (2025). Artificial intelligence and stroke imaging. Curr. Opin. Neurology 38, 40–46. 10.1097/wco.0000000000001333 PMC11706347 39760722

[B111] Rorden C. McCormick M. Hanayik T. Masoud M. Plis S. M. (2025). brain2print AI powered web tool for creating 3D printable brain models. Sci. Rep. 15, 15664. 10.1038/s41598-025-00014-5 40325035 PMC12053561

[B112] Saiz-Vivó M. Mill J. Iriart X. Cochet H. Piella G. Sermesant M. (2025). Digital twin integrating clinical, morphological and hemodynamic data to identify stroke risk factors. NPJ Digital Medicine 8, 369. 10.1038/s41746-025-01676-1 40527917 PMC12174328

[B113] Sar A. Puri P. S. Naz H. Aich S. Choudhury T. Gabralla L. A. (2025). Multi-modal deep learning framework for early detection of Parkinson’s disease using neurological and physiological data for high-fidelity diagnosis. Sci. Rep. 15, 34835. 10.1038/s41598-025-21407-6 41057513 PMC12504576

[B114] Saxena S. Tsobgnie M. N. Südy R. Gisselbaek M. Lechien J. R. Carella M. (2026). Generation Z *versus* generative artificial intelligence: a cross-sectional study assessing medical students’ confidence and over-reliance on artificial intelligence in perioperative clinical scenarios. Eur. J. Anaesthesiol. 43, 273–275. 10.1097/eja.0000000000002282 40965849

[B115] Scholten K. Meng E. (2018). A review of implantable biosensors for closed-loop glucose control and other drug delivery applications. Int. J. Pharm. 544, 319–334. 10.1016/j.ijpharm.2018.02.022 29458204

[B116] Segato A. Marzullo A. Calimeri F. De Momi E. (2020). Artificial intelligence for brain diseases: a systematic review. Apl. Bioeng. 4, 041503. 10.1063/5.0011697 33094213 PMC7556883

[B117] Serag I. Azzam A. Y. Hassan A. K. Diab R. A. Diab M. Hefnawy M. T. (2025). Multimodal diagnostic tools and advanced data models for detection of prodromal Parkinson’s disease: a scoping review. BMC Medical Imaging 25, 103. 10.1186/s12880-025-01620-5 40155878 PMC11951780

[B118] Sharma S. Chhetry A. Zhang S. Yoon H. Park C. Kim H. (2021). Hydrogen-bond-triggered hybrid nanofibrous membrane-based wearable pressure sensor with ultrahigh sensitivity over a broad pressure range. ACS Nano 15, 4380–4393. 10.1021/acsnano.0c07847 33444498

[B119] Shen J. (2022). Plasticity of the central nervous system involving peripheral nerve transfer. Neural Plast. 2022, 5345269–10. 10.1155/2022/5345269 35342394 PMC8956439

[B120] Si Z.-Z. Zou C.-J. Mei X. Li X. F. Luo H. Shen Y. (2022). Targeting neuroinflammation in Alzheimer’s disease: from mechanisms to clinical applications. Neural Regen. Res. 18, 708–715. 10.4103/1673-5374.353484 PMC9700083 36204826

[B121] Sill M. Sahm F. Schrimpf D. Capper D. Pfister S. M. von Deimling A. (2020). Path-04. an enhanced ai-driven platform for precision molecular brain tumor dianostics. Neuro-Oncol. 22, iii425. 10.1093/neuonc/noaa222.640

[B122] Sizemore N. Oliphant K. Zheng R. Martin C. R. Claud E. C. Chattopadhyay I. (2024). A digital twin of the infant microbiome to predict neurodevelopmental deficits. Sci. Adv. 10, eadj0400. 10.1126/sciadv.adj0400 38598636 PMC11006218

[B123] Smith K. Brooks W. Challiner J. Povey E. (2021). Quality assurance of remote clinical assessments in the NHS. Lancet London, Engl. 398, 1962. 10.1016/s0140-6736(21)02328-x PMC8616570 34838172

[B124] Song S. Li T. Lin W. Liu R. Zhang Y. (2025). Application of artificial intelligence in Alzheimer’s disease: a bibliometric analysis. Front. Neurosci. 19, 1511350. 10.3389/fnins.2025.1511350 40027465 PMC11868282

[B125] Sriramulu V. Rajendran A. (2026). Avatar authenticated metaverse environment with improved key management and information flow control with obfuscation. Cybern. Syst. 57, 308–349. 10.1080/01969722.2025.2598613

[B126] Stein J. B. Zhang S. Roh E. J. Luo J. Chen M. Jang H. (2025). Advanced biomaterial delivery of hypoxia-conditioned extracellular vesicles (EVs) as a therapeutic platform for traumatic brain injury. Adv. Sci. 12, e04147. 10.1002/advs.202504147 PMC12667507 40919721

[B127] Stewart N. J. Sato T. Takeda N. Hirata H. Matsumoto S. (2022). Hyperpolarized 13C magnetic resonance imaging as a tool for imaging tissue redox State, oxidative stress, inflammation, and cellular metabolism. Antioxidants & Redox Signal. 36, 81–94. 10.1089/ars.2021.0139 PMC8792501 34218688

[B128] Suvarna M. Usteri M. E. Krumeich F. Mitchell S. Pérez‐Ramírez J. (2025). Explainable artificial intelligence elucidates synthesis-structure-property-function relationships in nanostructured catalysts. Adv. Mater. 37, e2420465. 10.1002/adma.202420465 40370133

[B129] Tablante J. Casaletto K. VandeVrede L. Mamuyac E. Gao L. Esteban J. S. (2025). The role of bilingualism on functional decline and neurodegeneration in distinct ADRD clinical syndromes. Alzheimer’s & Dementia J. Alzheimer’s Assoc. 21, e70991. 10.1002/alz.70991 PMC12715707 41416535

[B130] Takahashi Y. Idei H. Komatsu M. Tani J. Tomita H. Yamashita Y. (2025). Digital twin brain simulator for real-time consciousness monitoring and virtual intervention using primate electrocorticogram data. NPJ Digital Medicine 8, 80. 10.1038/s41746-025-01444-1 39929926 PMC11811282

[B131] Takeuchi H. Suwa K. Kishi A. Nakamura T. Yoshiuchi K. Yamamoto Y. (2022). The effects of objective push-type sleep feedback on habitual sleep behavior and momentary symptoms in daily life: mhealth intervention trial using a health care internet of things system. JMIR mHealth uHealth 10, e39150. 10.2196/39150 36201383 PMC9585447

[B132] Takiyama K. Sakurada T. Shinya M. Sato T. Ogihara H. Komatsu T. (2020). Larger, but not better, motor adaptation ability inherent in medicated Parkinson’s disease patients revealed by a smart-device-based study. Sci. Rep. 10, 7113. 10.1038/s41598-020-63717-x 32346067 PMC7188883

[B133] Tavakoli N. Shakeri Z. Gowda V. Samsel K. Bedayat A. Ghasemiesfe A. (2025). Generative AI and foundation models in radiology: applications, opportunities, and potential challenges. Radiology 317, e242961. 10.1148/radiol.242961 41251552

[B166] Thamizharasi M. Lakshmi M. (2026). Optimizing Alzheimer’s disease detection with dual attention CNN and gooseneck barnacle search. Cybern. Syst. 57, 225–258. 10.1080/01969722.2025.2521703

[B134] Therriault J. Gauthier S. Rosa-Neto P. (2023). Applications of Alzheimer’s disease staging to clinical trials. Aging (Albany NY) 15, 4–5. 10.18632/aging.204482 36622284 PMC9876645

[B135] Tonello S. Rolla R. Tillio P. A. Sainaghi P. P. Colangelo D. (2025). Microenvironment and tumor heterogeneity as pharmacological targets in precision oncology. Pharmaceuticals 18, 915. 10.3390/ph18060915 40573308 PMC12195999

[B136] Topalidis P. I. Demarchi G. Reisinger L. Schubert J. Ameen M. S. Weisz N. (2025). Selective preservation of prediction-related signals in human sleep. Curr. Biology CB 35, 2195–2201.e4. 10.1016/j.cub.2025.03.064 40245865

[B137] Topol E. J. (2020). Welcoming new guidelines for AI clinical research. Nat. Med. 26, 1318–1320. 10.1038/s41591-020-1042-x 32908274

[B138] Vallée A. (2026). Simulation-based model of menopause: hormonal changes and cardiovascular risk in a digital twin view. Climacteric J. Int. Menopause Soc. 29, 239–247. 10.1080/13697137.2025.2567693 41182000

[B139] Varma V. R. Desai R. J. Navakkode S. Wong L. W. Anerillas C. Loeffler T. (2023). Hydroxychloroquine lowers Alzheimer’s disease and related dementias risk and rescues molecular phenotypes related to Alzheimer’s disease. Mol. Psychiatry 28, 1312–1326. 10.1038/s41380-022-01912-0 36577843 PMC10005941

[B140] Vinci-Booher S. Ren X. Kay K. Yu C. Pestilli F. Booth J. R. (2026). Dense longitudinal neuroimaging reveals individual brain change trajectories. Trends Cognitive Sci. 30, 464–476. 10.1016/j.tics.2025.09.005 PMC12667233 41058412

[B141] Visvanathan A. Graham C. Dennis M. Lawton J. Doubal F. Mead G. (2021). Predicting specific abilities after disabling stroke: development and validation of prognostic models. Int. J. Stroke 16, 935–943. 10.1177/1747493020982873 33402051 PMC8554496

[B142] Vlachakis D. Papakonstantinou E. Sagar R. Bacopoulou F. Exarchos T. Kourouthanassis P. (2021). Improving the utility of polygenic risk scores as a biomarker for Alzheimer’s disease. Cells 10, 1627. 10.3390/cells10071627 34209762 PMC8305482

[B143] Wang L. Yang G. (2025). ViT-BiLSTM multimodal learning for paediatric ADHD recognition: integrating wearable sensor data with clinical profiles. Sensors 25, 6459. 10.3390/s25206459 41157512 PMC12567585

[B144] Wang X. Mao W. Wang Z. Li X. Xiong Y. Lu H. (2020). Enhanced anti-brain metastasis from non-small cell lung cancer of osimertinib and doxorubicin Co-Delivery targeted nanocarrier. Int. J. Nanomedicine 15, 5491–5501. 10.2147/IJN.S258699 32848385 PMC7425109

[B145] Wen J. (2025). Towards a multi-organ, multi-omics medical digital twin. Nat. Biomed. Eng. 9, 1386–1389. 10.1038/s41551-025-01474-w 40855122

[B146] Wilms L. K. Lossius M. I. Annala K. Abdel‐Khalik J. Fanter L. Elomaa K. (2026). Seizure classification using a multimodal seizure monitoring system (Nelli) in Dravet and Lennox-Gastaut syndromes: a non-randomized, single-center feasibility study. Epilepsia 67, 164–174. 10.1111/epi.18640 41066202 PMC12893256

[B147] Xiang G. Yin B. Shiroud Heidari B. Youssef G. Gosecka M. Gosecki M. (2025). Programmable hydrogels: frontiers in dynamic closed-loop systems, biomimetic synergy, and clinical translation. Adv. Sci. 12, e12037. 10.1002/advs.202512037 PMC12713069 41235760

[B148] Xie J. Wu Q. Dey N. Shi F. Sherratt R. S. Kuang Y. (2025). Empowering stroke recovery with upper limb rehabilitation monitoring using TinyML based heterogeneous classifiers. Sci. Rep. 15, 18090. 10.1038/s41598-025-01710-y 40413260 PMC12103544

[B149] Xu X. Lin M. Xu T. (2022). Epilepsy seizures prediction based on nonlinear features of EEG signal and gradient boosting decision tree. Int. J. Environ. Res. Public Health 19, 11326. 10.3390/ijerph191811326 36141613 PMC9517630

[B150] Xu S. Kantarcigil C. Rangwala R. Nellis A. San Chun K. Richards D. (2025). Digital health technology for Parkinson’s disease with comprehensive monitoring and artificial intelligence-enabled haptic biofeedback for bulbar dysfunction. J. Parkinson’s Dis. 15, 630–645. 10.1177/1877718x251329354 40183358 PMC13347456

[B151] Yan J. Zhao Y. Chen Y. Wang W. Duan W. Wang L. (2021). Deep learning features from diffusion tensor imaging improve glioma stratification and identify risk groups with distinct molecular pathway activities. EBioMedicine 72, 103583. 10.1016/j.ebiom.2021.103583 34563923 PMC8479635

[B152] Yan W. Lin Y. Chen Y.-F. Wang Y. Wang J. Zhang M. (2025). Enhancing neuroplasticity for post-stroke motor recovery: mechanisms, models, and neurotechnology. IEEE Transactions Neural Systems Rehabilitation Engineering A Publication IEEE Eng. Med. Biol. Soc. 33, 1156–1168. 10.1109/tnsre.2025.3551753 40100694

[B153] Young J. S. Morshed R. A. Gogos A. J. Amara D. Villanueva-Meyer J. E. Berger M. S. (2020). The glioma-network interface: a review of the relationship between glioma molecular subtype and intratumoral function. Neurosurgery 87, 1078–1084. 10.1093/neuros/nyaa362 34791466 PMC7666965

[B154] Zaccagna F. Grist J. T. Quartuccio N. Riemer F. Fraioli F. Caracò C. (2021). Imaging and treatment of brain tumors through molecular targeting: recent clinical advances. Eur. J. Radiology 142, 109842. 10.1016/j.ejrad.2021.109842 34274843

[B155] Zeng N. Zuo S. Zheng G. Ou Y. Tong T. (2020). Editorial: artificial intelligence for medical image analysis of neuroimaging data. Front. Neurosci. 14, 480. 10.3389/fnins.2020.00480 32508575 PMC7253661

[B156] Zeng X. Ahmed A. Tunio M. H. (2025). A balanced multimodal multi-task deep learning framework for robust patient-specific quality assurance. Diagnostics 15, 2555. 10.3390/diagnostics15202555 41153228 PMC12564269

[B157] Zhang Q. Sun D. (2025). Micro/nanorobots for combating brain disorders: challenges, advances, and perspectives. Adv. Sci. 12, e16592. 10.1002/advs.202516592 PMC12667542 41103256

[B158] Zhang C. Yang Y. Han S. Xu L. Chen X. Geng X. (2022). The temporal dynamics of large‐scale brain network changes in disorders of consciousness: a microstate-based study. CNS Neurosci. & Ther. 29, 296–305. 10.1111/cns.14003 36317719 PMC9804064

[B159] Zhang H. Fernando I. R. Han J. Nguyen K. T. Liu J. L. (2022). Editorial: advanced self-assembled materials with programmable functions. Front. Chem. 10, 892461. 10.3389/fchem.2022.892461 35518714 PMC9062694

[B160] Zhang M. Riddle J. Frohlich F. (2022). Closed-loop control of bistable symptom states. Brain Stimulation 15, 454–456. 10.1016/j.brs.2022.02.010 35219926 PMC9286699

[B161] Zhang J. Du Y. Li J. Yang W. Cao D. Luo N. (2025). Stage-dependent neural mechanisms in human methamphetamine abstinence: insights from the digital twin brain model. Biol. Psychiatry 98, 634–645. 10.1016/j.biopsych.2025.05.010 40403824

[B162] Zhang R. Aksman L. Wijesinghe D. Ringman J. M. Wang D. J. J. Jann K. (2025). A longitudinal study of functional brain complexity in progressive Alzheimer’s disease. Alzheimer’s & Dementia 17, e70059. 10.1002/dad2.70059 PMC11736706 39822290

[B163] Zhao Y. Wang B. Hojaiji H. Wang Z. Lin S. Yeung C. (2020). A wearable freestanding electrochemical sensing system. Sci. Adv. 6, eaaz0007. 10.1126/sciadv.aaz0007 32219164 PMC7083607

[B164] Zheng Z. Zhu R. Peng I. Xu Z. Jiang Y. (2024). Wearable and implantable biosensors: mechanisms and applications in closed-loop therapeutic systems. J. Mater. Chem. B 12, 8577–8604. 10.1039/d4tb00782d 39138981

[B165] Zheng B. Tang J. Nie C. Jiang Y. Dimde M. Li W. (2026). Oxidized cellulose nanofibril hydrogel as a 3D scaffold for breast tumor spheroid expansion and microenvironment modeling. Int. J. Biol. Macromol. 338, 149716. 10.1016/j.ijbiomac.2025.149716 41412241

