# Supplementary material for: From fragmented neurotechnologies to closed-loop brain health systems: integrating artificial intelligence, digital health, digital twins, and advanced materials for brain disorders
Source: Front Bioeng Biotechnol. 2026 Aug 7;14:1904561. doi: 10.3389/fbioe.2026.1904561 (PMC13493567; doi:10.3389/fbioe.2026.1904561)
Supplement: Supplementary file 1 [file Supplementaryfile1.docx]

## **Supplementary Table 1. Conventional fragmented model vs closed-loop brain health system**

| Dimension | Conventional fragmented model | Closed-loop brain health system | Translational implication |
| --- | --- | --- | --- |
| Core logic | Diagnosis, monitoring, treatment, and follow-up are handled as separate clinical stages | Diagnosis, monitoring, simulation, intervention, and feedback are integrated into a continuous adaptive cycle | Moves brain disease care from episodic management to dynamic disease-state management |
| Disease representation | Disease is mainly represented by diagnostic labels or isolated clinical scores | Disease is represented as a dynamic brain state shaped by biological, functional, behavioral, and environmental data | Enables more precise stratification, prognosis, and intervention timing |
| Data structure | Cross-sectional imaging, clinic-based examinations, and occasional laboratory results | Longitudinal multimodal data from imaging, electrophysiology, omics, wearables, smartphones, clinical records, and patient-reported outcomes | Improves sensitivity to progression, fluctuation, recovery, and relapse |
| Role of artificial intelligence | Automated detection, segmentation, classification, or risk prediction for isolated tasks | Brain-state interpretation, trajectory modeling, decision support, and integration of heterogeneous data | Shifts AI from diagnostic automation to clinical decision architecture |
| Role of digital health | Remote data collection or symptom monitoring | Therapeutic infrastructure connecting patients, clinicians, algorithms, and interventions | Converts monitoring into actionable feedback and adaptive care |
| Role of digital biomarkers | Device-derived measurements used as exploratory signals | Clinically interpretable and biologically anchored markers linked to decisions | Requires validation of technical reliability, clinical validity, and clinical utility |
| Role of digital twins | Often conceptual or used as standalone predictive models | Patient-specific, dynamically updated simulation layer for disease trajectory and intervention response | Supports individualized treatment planning and adaptive follow-up |
| Role of advanced materials | Passive carriers, scaffolds, or delivery vehicles | Programmable therapeutic interfaces responding to disease states and integrated with monitoring | Aligns biomaterial therapy with timing, localization, safety, and functional outcomes |
| Therapeutic strategy | Treatment decisions are often based on guidelines, clinician judgment, and periodic reassessment | Interventions are adjusted according to changing patient state, predicted risk, and feedback data | Supports personalized pharmacology, rehabilitation, neuromodulation, surgery, and material-mediated therapy |
| Evaluation endpoints | Accuracy, sensitivity, specificity, drug loading, release profile, or short-term biomarker change | Functional outcome, longitudinal benefit, workflow integration, safety, usability, cost-effectiveness, and equity | Promotes system-level translational evaluation rather than single-device performance |
| Clinical workflow | Technologies are added onto existing workflows, sometimes increasing burden | Technologies are co-designed with clinical pathways, electronic health records, and care teams | Improves adoption, sustainability, and clinician trust |
| Patient role | Patient is often a passive recipient of tests and interventions | Patient and caregiver participate in monitoring, reporting, decision-making, and feedback | Enhances patient-centered care but requires attention to burden and accessibility |
| Governance | Privacy, consent, bias, and accountability are addressed after technology development | Governance is built into system design from the beginning | Improves trust, regulatory readiness, and ethical deployment |
| Main risk | Fragmented innovations may remain technically impressive but clinically disconnected | Integrated systems may become complex, intrusive, or inequitable if poorly governed | Requires balanced design, rigorous validation, and patient-centered implementation |
| Ideal outcome | Better individual tools for diagnosis, monitoring, or therapy | Adaptive, interpretable, clinically deployable, and patient-specific brain health management | Establishes a pathway toward closed-loop precision neurology |
